# Limited impact of *Salmonella* stress and persisters on antibiotic clearance

**DOI:** 10.1038/s41586-024-08506-6

**Published:** 2025-02-05

**Authors:** Joseph Fanous, Beatrice Claudi, Vishwachi Tripathi, Jiagui Li, Frédéric Goormaghtigh, Dirk Bumann

**Affiliations:** Biozentrum, University of Basel, Basel, Switzerland

**Keywords:** Antibiotics, Pathogens

## Abstract

Antimicrobial compounds are essential for controlling bacterial infections. Stress-induced bacterial tolerance and persisters can undermine antimicrobial activities under laboratory conditions, but their quantitative effects under physiological conditions remain unclear^1,2^. Here we determined constraints on clearance of *Salmonella* by antimicrobials in infected mice and tissue-mimicking chemostats. The antibiotics enrofloxacin and ceftriaxone exhibited poor anti-*Salmonella* activity under both conditions, primarily owing to severe nutrient starvation, which restricted *Salmonella* replication^3–5^. Other infection-associated conditions, such as acidic pH, glucose, oxidative stress, nitrosative stress, antimicrobial peptides, osmolarity, oxygen limitation, carbon dioxide and carbonate, as well as drug efflux, toxin–antitoxin modules and cell size had limited effects. A subset of resilient *Salmonella* appeared as a key obstacle for clearance by enrofloxacin, based on the biphasic decline of *Salmonella* colony-forming units. However, these data were misleading, because colony formation was confounded by extensive post-exposure killing. More accurate single-cell, real-time assays showed uniformly slow damage, indicating high resilience across the entire *Salmonella* population. The resulting extensive survival of bulk bacteria minimized the effect of hyper-resilient persisters. Thus, starvation-induced general resilience of *Salmonella* was the main cause of poor antibiotic clearance. These findings highlight the importance of quantifying antibiotic activity with real-time, single-cell assays under physiological conditions.

## Abstract (web summary)

Experiments in infected mice and tissue-mimicking chemostats show that resilience of *Salmonella* against antimicrobial compounds is largely a result of starvation-induced general resilience rather than survival of a small subset of hyper-resilient cells.

## Main

Effective antimicrobial therapy is crucial for control of bacterial infections. Antimicrobial resistance can impede treatment success, but eradication failures occur even in the absence of resistance^6^. These failures are often attributed to host stresses that trigger bacterial drug tolerance^1^ and/or subsets of bacteria, such as persisters, that are refractory to killing^2^. Supporting evidence comes mostly from laboratory conditions, and the relevance under physiological conditions remains unclear. Here we determined the quantitative effect of bacterial stresses and heterogeneity on antimicrobial efficacy in a mouse model of invasive salmonellosis, a difficult-to-treat infection that affects around 15 million patients and causes around 200,000 deaths annually^7^. Our mouse model replicates treatment failures observed in humans^8^ and exposes *Salmonella* to diverse stresses, leading to the formation of heterogeneous bacterial subsets with divergent properties and fates^9,10^. Thus, the model is suitable for assessing the effects of host stresses and bacterial heterogeneity on antimicrobial clearance in a clinically relevant context.

## Inefficient killing of *Salmonella*

**Fig. 1:**
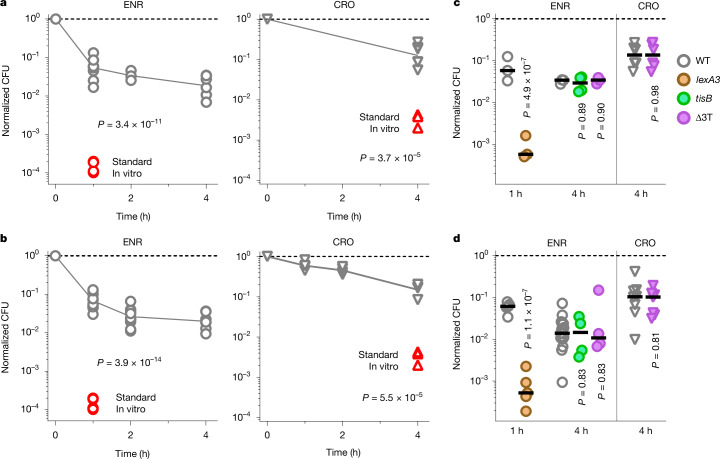
Poor anti-*Salmonella* activities of enrofloxacin and ceftriaxone. **a**, *Salmonella* survival in mouse spleen after an antibiotic dose (grey symbols show re-analysed data from ref. ^12^; enrofloxacin (ENR): 1 h, *n* = 10; 2 h, *n* = 3; 4 h, *n* = 6; ceftriaxone (CRO), *n* = 6) or in Mueller–Hinton broth under normal atmosphere at 37 °C (red symbols (this study); enrofloxacin, *n* = 5; ceftriaxone, *n* = 3). Each symbol represents an individual mouse or an independent in vitro culture. Lines connect the geometric means. Two-tailed *t*-test of log-transformed data. **b**, *Salmonella* survival in tissue-mimicking chemostat cultures (grey symbols show re-analysed data from ref. ^12^; enrofloxacin: 1 h and 2 h, *n* = 12; 4 h, *n* = 7; ceftriaxone 1 h, 2 h and 4 h, *n* = 4) or laboratory conditions (red symbols; number of samples as in **a**). Each symbol represents an individual chemostat reactor. Lines connect the geometric means. Two-tailed *t*-test of log-transformed data. **c**, Survival of wild-type (WT) and indicated mutant *Salmonella* in spleen. Each symbol represents an individual mouse (enrofloxacin: wild type and *lexA3* 1 h, *n* = 3; wild type 4 h, *n* = 3; *tisB*, *n* = 4; Δ3T, *n* = 3; ceftriaxone: wild type and Δ3T, *n* = 6). Horizontal bars represent geometric means. Enrofloxacin, one-way ANOVA of log-transformed data with comparisons to wild-type data and Holm–Šídák correction for multiple comparisons; ceftriaxone, two-tailed *t*-test of log-transformed data. **d**, Survival of wild-type and indicated mutant *Salmonella* in chemostat cultures. Each symbol represents an individual chemostat reactor (enrofloxacin: wild type and *lexA3* 1 h, *n* = 5; wild type 4 h, *n* = 16; *tisB* and Δ3T, *n* = 4; ceftriaxone: wild type, *n* = 8; Δ3T, *n* = 7). Horizontal bars represent geometric means (statistical tests as in **c**). Source data

We infected genetically susceptible mice with *gfp*-expressing *Salmonella enterica* subsp. *enterica* serovar Typhimurium. Upon appearance of clinical symptoms, we administered the recommended dose of 5 mg per kg (body weight) enrofloxacin (the widely used veterinary version and prodrug of ciprofloxacin), which is distributed organism-wide^11^ within approximately 10 min. After 1 h, we euthanized the mice and recovered an approximately 20-fold diminished number of *Salmonella* colony-forming units (CFUs) from the major target organ spleen^12^. This CFU loss was around 300-fold slower compared with standard laboratory conditions for antimicrobial susceptibility testing (exponential growth in Mueller–Hinton broth under normoxia at 37 °C) with an equivalent enrofloxacin concentration^12^ (1.5  mg l^−1^) (Fig. 1a). Similarly, a dose of 50 mg kg^−1^ ceftriaxone diminished CFUs in spleen approximately 8-fold over 4 h, around 40-fold slower than under standard laboratory conditions with 25 mg l^−1^ ceftriaxone. Thus, the two recommended bactericidal antibiotic classes for treatment of invasive salmonellosis (fluoroquinolones and cephalosporins)^7^ had poor anti-*Salmonella* activity in infected mice, consistent with slow antimicrobial clearance in humans^13^. This was not owing to the emergence of resistance, because *Salmonella* from treated mice^12^ and humans retain full susceptibility. Slow clearance is the primary reason for eventual treatment failures because some *Salmonella* still survive in host tissues when the critical support of host inflammation vanishes^12^.

## Limited effect of diverse stresses

To identify constraints on anti-*Salmonella* activities, we mimicked the extensively characterized *Salmonella* tissue microenvironments in chemostats. We used 10% oxygen, 5% carbon dioxide balanced with 10 mM bicarbonate at pH 5.6, and 23 nutrients available to *Salmonella* in the mouse spleen^5^. We set the inflow of fresh medium to 0.116 ml h^−1^ per ml of culture, mimicking the severe nutrient starvation that limits *Salmonella* growth in mice^3^ (approximately 0.16 divisions per hour, generation time approximately 6 h). Under these conditions, physiological concentrations of enrofloxacin^12^ (1.5 mg l^−1^) or ceftriaxone^14^ (25 mg l^−1^) killed *Salmonella* at rates similar to those in mice (Fig. 1b). A *lexA3* allele, which blocks repair of DNA double-strand breaks (DSBs)^15^, accelerated enrofloxacin killing approximately 100-fold in mice and chemostats (Fig. 1c,d), indicating that enrofloxacin triggered DSBs (which are lethal without repair) in almost all *Salmonella*, consistent with pharmacological target attainment^12^ under both conditions. Toxins encoded by *tisB*^16^ or *ecnB*^3^, *shpAB* and *phD-doc*^17^ (deleted in Δ3T), had no detectable effects on enrofloxacin and ceftriaxone activities in mice and chemostats^18^ (Fig. 1c,d). In summary, tissue-mimicking chemostats recapitulated the poor in vivo activities of both antibiotics.

**Fig. 2:**
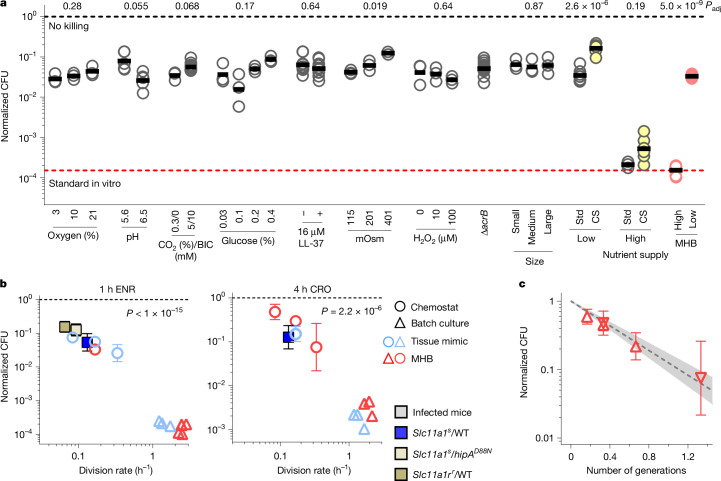
Modulation of *Salmonella* killing by stresses and nutrient supply. **a**, *Salmonella* survival after 1 h exposure to enrofloxacin in chemostat cultures with varying conditions. Each symbol represents an individual chemostat reactor. *P* values adjusted for multiple comparisons (Holm–Šídák) (two-tailed *t*-test on log-transformed data for pH; ANOVA on log-transformed data for glucose and osmolarity). BIC, bicarbonate; CS, combined stresses; MHB, Mueller–Hinton broth; Std, standard tissue-mimicking conditions. **b**, Survival of *Salmonella* in mice (squares, geometric mean ± geometric s.d.; enrofloxacin: *Slc11a1*^*s*^/WT, *n* = 10; *Slc11a1*^*s*^/*hipA*^*D88N*^, *n* = 4; *Slc11a1*^*r*^/WT, *n* = 8; ceftriaxone, *n* = 6), or in chemostats in tissue-mimicking medium (geometric mean ± geometric s.d.; enrofloxacin for division rates 0.083 h^−1^, 0.17 h^−1^ and 0.33 h^−1^, *n* = 10; ceftriaxone, *n* = 4), Mueller–Hinton broth (enrofloxacin or ceftriaxone for division rates 0.083 h^−1^, 0.17 h^−1^ and 0.33 h^−1^, *n* = 5) or in individual batch cultures (enrofloxacin: tissue-mimicking medium, *n* = 3; Mueller–Hinton broth, *n* = 5; ceftriaxone: tissue-mimicking medium and Mueller–Hinton broth, *n* = 3). One-way ANOVA with test for linear trend for log-transformed data for tissue-mimicking medium or Mueller–Hinton broth cultures. **c**, Relationship between exposure time to ceftriaxone to number of generations and *Salmonella* survival for different proliferation rates and exposure times (geometric mean and geometric s.d.; upward triangles, division rate 0.17 h^−1^ and exposure for 1 h (*n* = 4), 2 h (*n* = 4) or 4 h (*n* = 9); downward triangles, 4 h exposure and division rates 0.08 (*n* = 5) or 0.33 h^−1^ (*n* = 5)). The dashed line represents a monoexponential fit and the shaded area shows the 95% confidence interval. Source data

**Extended Data Fig. 1:**
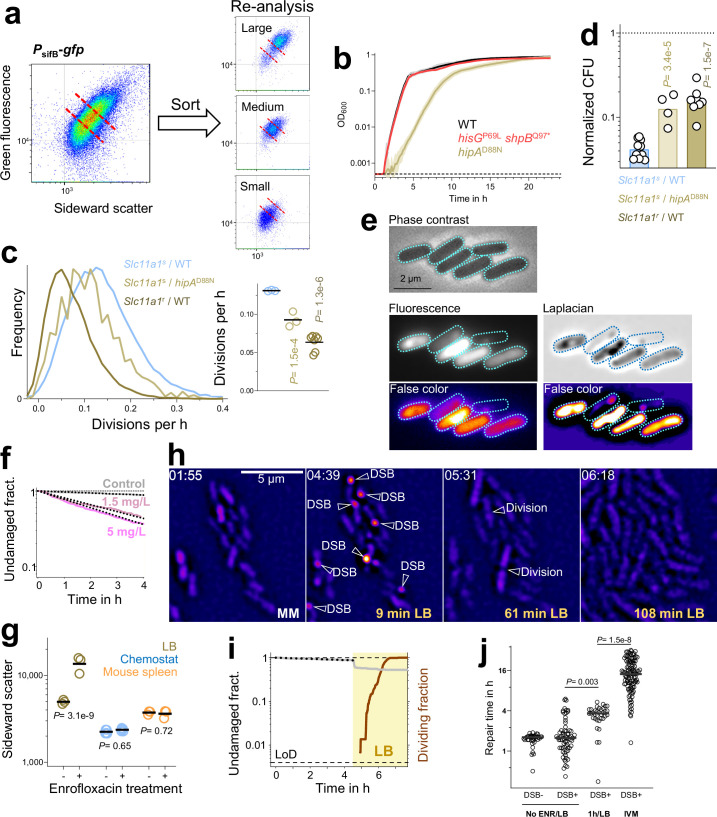
Analysis of *Salmonella* in vitro. (**a**) Sorting of *Salmonella* cells with different sizes. *Salmonella* expressing *gfp* under control of the chromosomal *P*_sifB_ promoter^61^ showed correlated sideward-scatter (a proxy for size^73^) and green fluorescence signals. The combination of the two detection channels increased resolution and enabled enrichment of subsets by flow cytometric sorting using the gate boundaries shown as dashed red lines. (**b**) Growth curves in lysogeny broth in 96-well plates. Means and SDs for 4 (wild-type, WT), 5 (*hipA*^D88N^), or 6 (*shpB*^Q97*^) biological replicates (each measured in technical triplicates) are shown (OD_600_, optical density at 600 nm; Det. Lim., detection limit). (**c**) Division rate distributions of *Salmonella* strains in different mouse genotypes (data for wild-type *Salmonella* in *SLC11A1*^r^ mice from^4^, n = 61,137 cells from seven independently infected mice; *SLC11A1*^s^, WT n = 34,851 from three independently infected mice; *SLC11A1*^s^, *hipA*^D88N^ n = 832 from three independently infected mice). The inset shows medians for the individual mice (*SLC11A1*^s^ WT/*hipA*^D88N^ n = 3; *SLC11A1*^r^, WT n = 7; ANOVA with comparison against *SLC11A1*^s^, WT; *P*-values adjusted for multiple comparisons according to Holm-Šídák). (**d**) Survival of *Salmonella* strains in mouse spleen 1 h after administration of 0.1 mg enrofloxacin. Each circle represents an individual mouse (*SLC11A1*^s^: WT n = 10; *hipA*^D88N^ n = 4; *SLC11A1*^r^, WT n = 7) two-tailed t-test, *P*-values adjusted for multiple comparisons according to Holm-Šídák). (**e**) Analysis of *Salmonella*/pRecA-mCherry fluorescence images. Detection of RecA-mCherry foci was enhanced by 2D Laplacian of Gaussian filtering (approximating a second derivative) of red fluorescence and false-color visualization. (**f**) Fraction of cells with no long-lasting RecA focus (“undamaged fraction”) during growth without enrofloxacin (Control, 146 cells), or exposure from t = 0 h to 1.5 or 5 mg/L enrofloxacin (130, 250 cells). The dotted lines represent monoexponential fits. The data for 5 mg/L are also shown in Fig. 3c. Summary data for the 5 mg/L exposure and an independent replicate are shown in Fig. 3d. (**g**) Flow-cytometry analysis of sideward scatter as a read-out for cell size^73^ of *Salmonella* before and after 1 h exposure to enrofloxacin during exponential growth in lysogeny broth (LB), slow growth in tissue-mimicking chemostats, or in mouse spleen. Each symbol represents an independent culture or an individual mouse (LB +/− n = 3; Chemostat +/− n = 5; Mouse spleen +/− n = 3; two-tailed t-test of log-transformed data with Holm-Šídák correction for multiple comparisons). (**h**) Snap-shots of *Salmonella* growing in nutrient-poor minimal medium (MM) without enrofloxacin and then in lysogeny broth (LB; video S1). (**i**) Fraction of undamaged cells before and after switching to LB, and dividing *Salmonella* after the LB switch (146 cells; LoD, limit of detection). RecA foci during the first 4 h are also shown in panel **f** (‘Control’). The dotted line is a monoexponential fit for damage before the LB switch. (**j**) Time difference between detection of a DNA double-strand bread (DSB) based on a long-lasting RecA focus, and re-initiation of cell division. Control cells without enrofloxacin exposure that were switched to lysogeny broth (No ENR/LB: DSB- n = 40; DSB+ n = 69), cells exposed for 1 h to enrofloxacin followed by a switch to LB (1 h/LB n = 35), and cells exposed to declining concentrations of enrofloxacin over 7 h followed by continuing incubation in nutrient-poor medium (‘*in vivo*-mimicking’, IVM n = 115) are shown. Each circle represents an individual cell. The statistical difference between groups was test using the Kruskal-Wallis-test with Dunn’s correction for multiple testing. Source data

To identify factors that constrained antibiotic activity in the chemostat experiments, we varied tissue-associated stresses that are known to affect fluoroquinolone efficacy, including acidic pH, reduced oxygen tension, increased carbon dioxide tension and bicarbonate concentration, osmolarity, glucose, oxidative stress and membrane-damaging antimicrobial peptides (Supplementary Note 1). We also inactivated AcrB, the main efflux pump for fluoroquinolones^19^ and a proposed cause of delayed antimicrobial clearance^20^, and compared *Salmonella* cells with different cell size or cell age^21^ (Extended Data Fig. 1a). Some of these parameters affected *Salmonella* killing by enrofloxacin, but the effects were generally small (up to threefold; glucose has an approximately sixfold effect) compared with the approximately 300-fold difference from standard laboratory conditions (Fig. 2a), consistent with their moderate effects under other in vitro conditions (Supplementary Note 1). Oxidative and nitrosative stresses were not required for poor enrofloxacin and ceftriaxone activities, consistent with their minor effects in vitro (Supplementary Note 1) and in mice (Supplementary Note 2). Combining conditions that promote survival (pH 5.6; 5% CO_2_/10 mM bicarbonate; 0.4% glucose; 401 mOsm) resulted in approximately fivefold greater survival (Fig. 2a, yellow circles). Unknown stresses or stress combinations may exist in vivo, but our results indicate the presence of a non-stress factor that dominates the high in vivo-like *Salmonella* survival in chemostats.

To determine the role of nutrient access, we grew *Salmonella* in low-density batch cultures, keeping all other parameters the same as in tissue-mimicking chemostats. Facile nutrient access under these conditions led to approximately tenfold faster replication (around 1.5 divisions per hour) and increased killing by enrofloxacin and ceftriaxone by about 200-fold (Fig. 2a,b), highlighting the critical role of nutrient access in antibiotic killing. Multiple linear regression of the data shown in Fig. 2a, with survival as dependent variable and nutrient access (which controlled the replication rate), oxygen, pH, CO_2_, glucose, LL-37, osmolarity and H_2_O_2_ as independent variables, explained 90.4% (*R*^2^) of the variance in survival (Extended Data Table 1). Nutrient access and replication rate (*P* < 10^−15^), osmolarity (*P* = 8.6 × 10^−7^) and H_2_O_2_ (*P* = 0.020) had a significant effect; of these factors, nutrient access and replication rate had by far the largest effect size (165-fold versus 3-fold (osmolarity) and 1.5-fold (H_2_O_2_); Fig. 2a). Thus, nutrient access controlling replication rate dominated antibiotic survival.

To confirm this, we starved *Salmonella* under non-physiological stress-free conditions (Mueller–Hinton broth, pH 7.4, normoxia) in chemostats, limiting *Salmonella* nutrition by slow medium inflow to achieve ~0.16 divisions per hour, as seen in vivo^3^. These conditions resulted in high *Salmonella* survival (approximately 3% after 1 h of enrofloxacin and 30% after 4 h of ceftriaxone; Fig. 2a–c) as in mice, indicating that starvation at levels similar to those experienced by *Salmonella* in mice^3,5^ was sufficient to achieve in vivo-like antibiotic survival rates, even under non-physiological, stress-free conditions. This was consistent with previous evidence linking starvation and slow replication with increased resilience against antibiotic activity^18,22^.

To determine the effect of replication rate in mice, we used two different approaches. First, we expressed the *Escherichia coli* toxin gene *hipA* in *Salmonella*. HipA inhibits translation and replication by phosphorylating GltX, but this can be prevented by the antitoxin HipB^23^. When HipA and HipB levels are similar, fluctuations create a bimodal distribution of growing ([HipA] < [HipB]) and non-growing ([HipA] > [HipB]) cells^23^. Without HipB, HipA impairs growth but does not create this heterogeneity. We expressed the partially detoxified allele^24^ *hipA*^*D88N*^ in *Salmonella* (which naturally lacks *hipAB*). This did not affect the enrofloxacin mean inhibitory concentration (MIC) (0.06 mg l^−1^), but reduced the replication rate in vitro (to around 47%) and in vivo (to around 71%; Extended Data Fig. 1b,c; competitive index in mice at day 4 post-infection, 0.028 ± 0.009, *n* = 6, *P* = 4 × 10^−5^, one-sample *t*-test of log-transformed data). In mice, *Salmonella hipA*^*D88N*^ survived enrofloxacin exposure around threefold better than parental *Salmonella* (Fig. 2b and Extended Data Fig. 1d). In a second approach, we tracked *Salmonella* killing in *Slc11a1*^*r*^ mice, which encode an active metal transporter SLC11A1 (also known as NRAMP1)^4^. SLC11A1 deprives *Salmonella* of magnesium, reducing its replication rate to around 50% of that in standard laboratory mice (*Slc11a1*^*s*^) with dysfunctional SLC11A1 (Extended Data Fig. 1c) without affecting stress levels or access to most other nutrients^4^. In *Slc11a1*^*r*^ mice infected with wild-type *Salmonella*, we observed around 15% CFU recovery after 1 h enrofloxacin treatment (approximately fourfold more than in *Slc11a1*^*s*^ mice; Fig. 2b and Extended Data Fig. 1d). Plating on low-nutrient minimal medium—which improves CFU recovery (see below)—resulted in a 1 h survival rate of about 32% (*n* = 4) in *Slc11a1*^*r*^ mice. This high survival is physiologically relevant because humans and wild mice carry *Slc11a1*^*r*^. Thus, slowing *Salmonella* replication in vivo using two different methods significantly increased antibiotic survival consistent with the growth–killing relationship observed in vitro.

These data indicate that the scarcity of carbon and energy sources (standard laboratory *Slc11a1*^*s*^ mice) and magnesium (in *Slc11a1*^*r*^ mice), which limit *Salmonella* replication^4,5^, are the primary cause of *Salmonella* resilience to antibiotics in vivo. This resilience could be owing to reduced DNA replication and cell wall synthesis (the targets of fluoroquinolones and cephalosporins) at slow growth and/or metabolic effects of limited nutritients^25^. Further research is required to disentangle these mechanisms.

## Biphasic loss of CFUs

Ceftriaxone killed *Salmonella* continuously by 0.90 ± 0.14 log per generation (Fig. 2c), suggesting slow loss of viability across the entire *Salmonella* population^26^. By contrast, enrofloxacin killed around 95% of *Salmonella* within the first 1 h of exposure (killing rate approximately 3 h^−1^) (Fig. 1a,b), and the remaining 5.4 ± 1.8% (in vivo) or 7.4 ± 3.3% (chemostat) lost viability at a slower rate over the next 3 h (killing rate 0.35 h^−1^). These biphasic kinetics suggested that a subset of 5–7% resilient *Salmonella* was a key obstacle for enrofloxacin-induced clearance in mice and in chemostats (antibiotic persistence)^2^.

According to consensus guidelines^2^, antibiotic persistence is measured by exposing bacteria to antibiotics for various time intervals, followed by washing and plating on agar plates. After overnight incubation, colonies are counted and the data are plotted against exposure time. A biphasic decline of CFUs (as observed here; Fig. 1a,b) is considered to be evidence that not all bacteria in the population are killed at the same rate. However, this strategy uses a late and indirect readout (colony formation overnight) to infer killing kinetics during antibiotic exposure, and relies on the assumption that each bacterium that is viable at the time of plating will form a colony. This assumption is violated in diverse pathogen–antibiotic combinations (Supplementary Note 3), indicating a need for more direct, real-time readouts of antimicrobial activity.

## Slow monophasic DNA damage

To monitor enrofloxacin activity in real-time, we detected DSBs, the crucial initial damage of fluoroquinolone action^27^, using *Salmonella* expressing a *recA*^*R29A*^-*mCherry* fusion. RecA–mCherry forms visible foci around all newly formed DSBs within <4 min (refs. ^28,29^) without interfering with subsequent DNA repair^29,30^. The R29A mutation prevents DSB-independent RecA aggregation^31^. Formation of RecA foci relies on the diffusion of constitutively present proteins with no need for de novo gene expression. RecA foci, therefore, serve as an early, sensitive, and reliable readout for DSBs before the initiation of SOS response and DNA repair^28–31^. Expressing *recA*^*R29A*^*-mCherry* from the *P*_recA_ promoter provides basal RecA–mCherry for visualizing emerging DSBs, and increasing RecA–mCherry levels during the subsequent SOS response (Extended Data Fig. 1e).

**Fig. 3:**
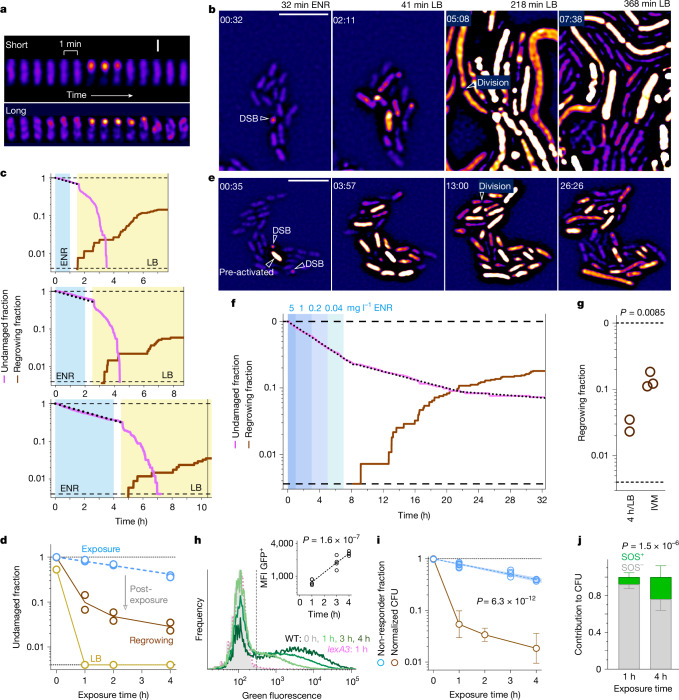
*Salmonella* DNA damage during and after exposure to enrofloxacin. **a**, Time-lapse gallery of RecA foci in enrofloxacin-exposed *Salmonella*. Scale bar, 1 μm. **b**, Snapshots of *Salmonella* exposed for 1 h to enrofloxacin, followed by drug washout for 30 min and incubation in LB. Scale bar, 5 μm. **c**, Fraction of undamaged and regrowing cells during 1 h (top), 2 h (middle) or 4 h (bottom) enrofloxacin exposure, washout and LB incubation (250 (top), 255 (middle) and 250 (bottom) cells; dotted lines show monoexponential fits for damage during exposure and washout; summary data and independent replicates in **d**,**g**). **d**, Fractions of undamaged cells at the end of enrofloxacin exposure, in LB, and regrowing survivors (two independent experiments, 755 and 1,233 cells). **e**, Snapshots of *Salmonella* exposed for 7 h to decreasing concentrations of enrofloxacin, followed by drug-free nutrient-poor medium (Supplementary Video 3). Scale bar, 5 μm. **f**, Fraction of undamaged and regrowing cells during and after 7 h exposure to decreasing concentrations of enrofloxacin (ENR) in nutrient-poor medium (276 cells; dotted lines show monoexponential fits for damage during exposure, post-antibiotic damage or baseline damage; summary data for this and two independent replicates in **g**). **g**, Fractions of regrowing cells after 4 h enrofloxacin exposure and a switch to LB (4 h/LB; 250 and 444 cells; same data as in **d**) or under in vivo-mimicking conditions (IVM) with declining enrofloxacin concentrations and regrowth in nutrient-poor medium (276, 271 and 378 cells). Circles represent independent experiments. Two-tailed *t*-test on log-transformed data. **h**, Fluorescence of *Salmonella*/pP_cad_-gfp or *Salmonella lexA3*/pP_cad_-gfp in untreated (0 h) or enrofloxacin-treated mice. The vertical line separates GFP^+^ responders from GFP^−^ non-responders. The inset shows the median fluorescence (MFI) of GFP^+^ cells, the line connects the geometric means (test for non-zero slope in a linear regression of log-transformed values). The histograms represent pooled data, circles in the inset graph represent individual mice (1 h, *n* = 5; 3 h and 4 h, *n* = 4). **i**, Blue, fraction of GFP^−^ non-responders in enrofloxacin-treated mice (0 h, *n* = 2; 1 h, *n* = 6; 2 h and 4 h, *n* = 4). Each symbol represents an individual mouse. Brown, CFUs recovered from similar samples (same data as in Fig. 1a; geometric mean ± geometric s.d.; 1 h, *n* = 10; 2 h, *n* = 3; 4 h, *n* = 6). Two-way ANOVA for difference between SOS response and CFU recovery. **j**, Contribution of responders (SOS^+^) and non-responders (SOS^−^) to CFU counts (Supplementary Note 5; data are mean ± s.d. for data from independently infected mice; 1 h, *n* = 6; 4 h, *n* = 4). Two-way ANOVA for difference between SOS^+^ and SOS^−^. Source data

We imaged *Salmonella* expressing *recA*^*R29A*^-*mCherry* in a microfluidic device during slow growth in tissue-mimicking, nutrient-poor medium. RecA–mCherry occasionally formed short-lived (less than 5 min) foci (Fig. 3a) indicating spontaneous, rapidly repairable damage during chromosomal replication^29^. Long-lasting RecA foci (lasting more than 5 min; Fig. 3a), indicating more persistent DSBs^28^, occurred at a rate of around 0.03 h^−1^ (Extended Data Fig. 1f), mostly without triggering a subsequent SOS response. These data were consistent with slow baseline DNA damage in untreated bacteria^29,31^.

Exposure to 1.5 mg l^−1^ enrofloxacin accelerated formation of long-lasting RecA foci to around 0.21 h^−1^ (Extended Data Fig. 1f). Each focus was followed by a SOS response, indicating severe DNA damage. The fraction of ‘undamaged’ cells (those that had not yet experienced any DSB), declined monoexponentially to around 40% after 4 h of enrofloxacin exposure. These real-time data were inconsistent with the CFU data because: (1) only around 15% cells experienced the critical primary DSB within 1 h of exposure, yet plating such cells showed a 20-fold decrease in CFU (Fig. 1a,b); and (2) damage was monoexponential, whereas the decrease in CFU was biexponential. The discrepancies were not explained by insufficient enrofloxacin exposure in the microfluidic device (for example, due to drug absorption in the microfluidic device), because increasing enrofloxacin concentration to 5 mg l^−1^ had only a minor effect on damage kinetics (Extended Data Fig. 1f), consistent with nearly saturating enrofloxacin exposure^12^ (we used 5 mg l^−1^ for all subsequent experiments). The discrepancies were not caused by filamentation of enrofloxacin-exposed bacteria, which might make them more prone to pipetting damage, because *Salmonella* in low-nutrient medium and in mice did not form filamented cells during enrofloxacin exposure (Extended Data Fig. 1g and Supplementary Video 2). The discrepancies were also not caused by insufficient sensitivity of DSB detection, because each SOS-responding cell had previously exhibited a long-lasting RecA focus, indicating comprehensive detection of relevant DNA damage^28,29,31^. RecA forms foci around all newly formed DSBs within minutes^28^, arguing against relevant delays in detection of the DNA damage. Thus, DNA damage during exposure was inconsistent with CFU data.

## Massive post-exposure damage

The discrepancies might arise during post-exposure regrowth on lysogeny broth (LB) agar plates until visible colonies form. We first assessed the effect of switching from nutrient-poor medium to LB without prior antibiotic exposure (simulating regrowth of untreated *Salmonella* on LB plates). Switching to LB triggered a burst of long-lasting RecA foci in around 40% of cells, suggesting substantial DNA stress in the initial 10 min after nutrient upshift (‘nutrient shock’^32^) (Extended Data Fig. 1h,i and Supplementary Video 1). However, only few of these events triggered SOS responses and around 98% of DSB^+^ cells restarted division after a lag of 1.5 h on LB in synchrony with undamaged DSB^−^ cells (Extended Data Fig. 1h–j and Supplementary Video 1), indicating efficient repair and negligible fitness loss.

We then simulated the entire CFU assay by exposing *Salmonella* for 1, 2 or 4 h to enrofloxacin in nutrient-poor medium, followed by drug washout for 30 min with antibiotic-free nutrient-poor medium, and regrowth in LB. Under these conditions, the shift to LB triggered vigorous RecA localization dynamics and SOS responses for several hours in all analysed cells, indicating severe DNA damage after the actual antibiotic exposure (Fig. 3b,c and Supplementary Video 2). Almost all cells increased in size, and many formed long filaments. Some cells reinitiated cell division about 3.5 h after the initial DSB (Extended Data Fig. 1j) and formed expanding microcolonies, indicating successful DNA repair. The frequency of these survivors (Fig. 3c,d, 1 h enrofloxacin exposure, about 10% survival; 4 h, about 3% survival) was similar to CFU data from enrofloxacin-treated chemostat cultures (Fig. 1b, 1 h, about 7% survival; 4 h, about 2% survival). Thus, enrofloxacin induced DSBs and killed *Salmonella* primarily post-exposure during regrowth on LB, explaining the discrepancy between limited damage during exposure and low CFU recovery.

Nutrient-rich LB is a poor mimic of relevant regrowth conditions in tissues. To approximate the in vivo situation, we grew *Salmonella* in nutrient-poor medium, exposed them to diminishing enrofloxacin concentrations approximating enrofloxacin pharmacokinetics in mice^11^, and maintained them in nutrient-poor medium after exposure. Under these conditions, *Salmonella* exhibited long-lasting RecA foci with monoexponential kinetics at 0.18 h^−1^ throughout the 7 h of enrofloxacin exposure and about 1 h after washout, followed by slower monoexponential kinetics at 0.07 h^−1^ for around 14 h in drug-free medium. Thereafter, focus formation returned to the basal rate of 0.02 h^−1^ (Fig. 3e,f and Supplementary Video 3). All cells with long RecA foci showed vigorous SOS responses and RecA localization dynamics indicating severe DNA damage. However, growth and filamentation were less extensive than in LB. Re-initiation of division and formation of microcolonies started 4 to 30 h after the initial DSB (Extended Data Fig. 1j), and around fivefold more cells survived than in 4 h exposure followed by LB regrowth (Fig. 3g), despite more damage at the end of exposure. Thus, scarce nutrition improved repair and survival, reaching levels (14 ± 4%; regrowing survivors) consistent with *Salmonella* loads in the spleen 24 h after enrofloxacin doses (34 ± 13% of pre-dose levels; this includes regrowing survivors and their daughter cells)^12^.

Together, our results demonstrate that enrofloxacin continued to damage *Salmonella* after the end of exposure. This was consistent with the half-life of ternary enrofloxacin–gyrase–DNA complexes of 2.5 h causing extended post-exposure growth inhibition (post-antibiotic effect)^33^. A shift to nutrient-rich LB caused extensive DSBs, explaining the need for DNA repair after, but not during, fluoroquinolone exposure in CFU assays^15,34^. Vigorous growth of enrofloxacin-poisoned cells on LB might cause biosynthetic imbalances that lead to viability loss^35^. Physiological starvation slowed regrowth^3,5^, resulting in less damage and allowing more time for repair.

**Extended Data Fig. 2:**
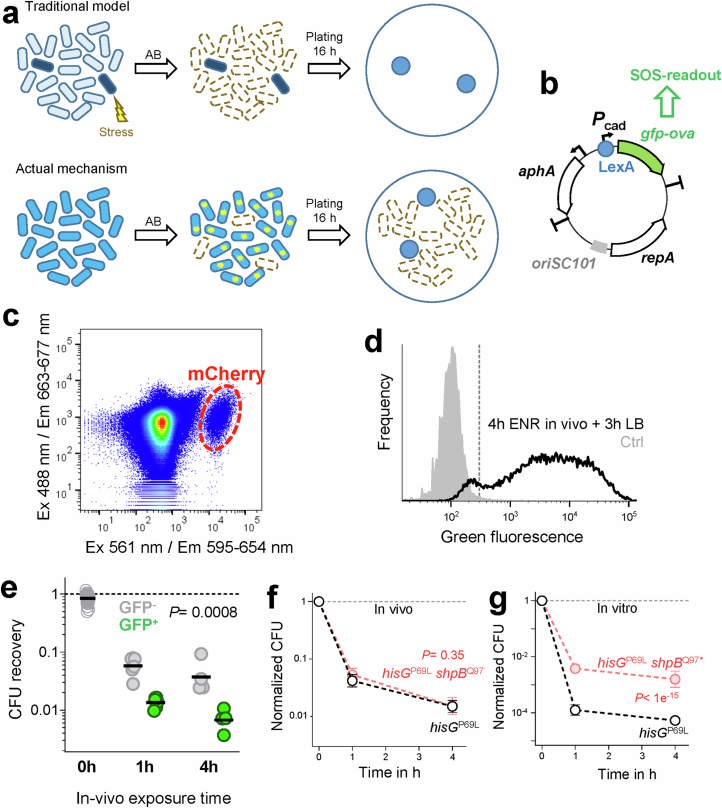
*Salmonella* analysis in vivo. (**a**) Traditional model and actual mechanism underlying rapid loss of colony-forming units. In the traditional models, a small subset of persisters, some of which are triggered by stresses, survive antibiotic exposure and give rise to colonies on plates. However, antibiotic exposure actually kills only few cells, but survivors are poisoned by drug, which remains bound to its target (depicted as yellow symbols). Most of these poisoned cells die after plating. Thus, colony counts reflect mostly post-exposure killing rather than viability loss during exposure. (**b**) Plasmid map of the SOS-reporter construct. The *P*_cad_ promoter is repressed by LexA^36^. LexA self-cleavage (SOS-response) is associated with unbinding of LexA to DNA, de-repressing *P*_cad_*. gfp-ova* encodes a destabilized GFP variant to report current promoter activities. *aphA* encodes aminoglycoside phosphotransferase conferring resistance to kanamycin. *repA* encodes RepA which replicates plasmids with a oriSC101 origin (T, terminator). (**c**) Gating strategy for identifying *Salmonella* reporter cells in spleen homogenates based on the red fluorescence of chromosomally encoded mCherry (Ex, excitation wavelength; Em, emission range). mCherry-*Salmonella* were then analyzed for green fluorescence as shown in Fig. 3h. (**d**) Green fluorescence of *Salmonella* retrieved from mouse spleen 4 h after enrofloxacin administration and incubated for 3 h in lysogeny broth (LB). Control *Salmonella* without *gfp* are also shown. The data represent histograms pooled from 2 mice. (**e**) Colony recovery of GFP^−^ (SOS^−^) and GFP^+^ (SOS^+^) *Salmonella* sorted from spleen homogenates from untreated mice (0 h), or from treated mice 1 h or 4 h after enrofloxacin administration. Survival was determined by plating on lysogeny-broth plates. Each circle represents an individual mouse (untreated n = 14; 1 h GFP +/− n = 6; 4 h GFP +/− n = 4). The statistical difference of GFP^-^ and GFP^+^ contributions from treated mice was tested by two-way ANOVA (column factor, two-tailed, single comparison). (**f**) Survival of *Salmonella* strains in enrofloxacin-treated mice (*hisG*^P69L^: 1 h n = 10; 4 h n = 6; *hisG*^P69L^ *shpB*^Q97*^ 1 h/4 h n = 3; geometric means and geometric SDs; two-way ANOVA of log-transformed data). (**g**) Survival of *Salmonella* strains in lysogeny broth with enrofloxacin (geometric means and geometric SDs; six independent cultures from two different experiments; two-way ANOVA of log-transformed data). Source data

These findings indicated that plotting CFU against exposure time was misleading, because most viability loss occurred not during exposure (as implicitly assumed in CFU-based assays), but after exposure. Thus, the approximately 20-fold drop in CFU on LB plates after 1 h of exposure did not represent the killing of 95% of the *Salmonella* population within that 1 h, and the gradual CFU decline with extended exposure did not indicate a refractory subpopulation. Instead, enrofloxacin exposure itself inflicted slow damage, but around 90% of the yet undamaged, enrofloxacin-poisoned *Salmonella* died post-exposure during regrowth, irrespective of prior exposure times (Fig. 3d and Extended Data Fig. 2a). Thus, the biphasic CFU kinetics did not reflect heterogeneous antibiotic survival but rather a confounded viability readout.

Real-time imaging revealed slow, monoexponential damage of *Salmonella* by enrofloxacin, suggesting low but uniform and constant damage probabilities for individual cells (akin to the radioactive decay of unstable atomic nuclei). Thus, the entire *Salmonella* population, and not just a small subset, was rather refractory to enrofloxacin-inflicted damage. Some damaged *Salmonella* were able to repair the DSBs and survived, further delaying clearance. We cannot exclude that some cells had lower damage probabilities or superior repair capabilities, but even the bulk population died at rates below 0.2 h^−1^.

## Slow monophasic SOS responses in mice

We next investigated *Salmonella* killing in mice. Detecting in vivo DNA damage by tracking RecA foci over hours is challenging in mouse tissues. Instead, we monitored the *Salmonella* SOS response to DNA damage using a reporter strain carrying a sensititve *P*_cad_-*gfp* fusion^36^ (Extended Data Fig. 2b,c). In the spleen of mice, the reporter showed negligible SOS activity before enrofloxacin administration (Fig. 3h), indicating limited host-induced DSBs (in contrast to infections of macrophages in cell culture; Supplementary Note 4).

Enrofloxacin administration activated around 25% *Salmonella* SOS reporter cells within 1 h (Fig. 3h,i). This was a specific SOS response, because SOS-defective *Salmonella lexA3* carrying the same reporter construct showed no response. Prolonged in vivo exposure to enrofloxacin increased the brightness and proportion of SOS^+^ *Salmonella* cells, resulting in a monoexponential decay of non-responders (SOS^−^) at a rate of 0.24 h^−1^. Non-responders retained a functional reporter construct and experienced enrofloxacin poisoning in vivo, because most reporter cells exhibited a SOS response post-exposure upon ex vivo incubation in LB^15^ (Extended Data Fig. 2d). Ex vivo sorting and plating of SOS^+^ and SOS^−^ *Salmonella* revealed that most colonies originated from SOS^−^ *Salmonella* cells that had not yet responded in vivo (Fig. 3j, Extended Data Fig. 2e and Supplementary Note 5). These observations were inconsistent with the CFU data because: (1) after 1 h of exposure only around 25% *Salmonella* responded to DNA damage, whereas the CFU dropped around 20-fold; (2) the response to DNA damage was monoexponential, whereas the CFU decline was biexponential; (3) most colonies originated from in vivo non-responders (SOS^−^), although an active SOS response was essential for formation of more than 99% of all colonies (based on the phenotype of SOS-deficient *Salmonella lexA3*; Fig. 1c). These data suggested that most DNA damage, SOS response and viability loss occurred after enrofloxacin exposure during regrowth on LB (Extended Data Fig. 2a), consistent with our observations in microfluidic devices (Fig. 3d).

To test the idea that regrowth on LB compromised the colony-forming ability of initially viable but enrofloxacin-poisoned *Salmonella*, we explored alternative plating media to reduce post-exposure damage. The addition of thiourea and dipyridiyl to mitigate oxidative stress had no effect on colony recovery, consistent with oxygen-independent killing by second-generation fluoroquinolones at concentrations achieved in vivo^12,37^. Lowering the incubation temperature diminished colony counts. Plating on the nutrient-poor medium used in the microfluidics devices yielded no visible colonies even after 7 days of incubation. However, after increasing carbon and energy sources tenfold, this medium supported formation of tiny colonies after 3 days at 37 °C. Plating spleen homogenates from enrofloxacin-treated mice on this medium yielded 2.2 ± 0.4-fold higher CFU than on LB (11 independent infections, *P* < 0.0001, two-tailed *t*-test on log-transformed values). This confirmed that a majority of enrofloxacin-poisoned but initially alive *Salmonella* died on LB plates, whereas some of them could repair the DNA damage and survive during slow regrowth on nutrient-poor medium^34^. The nutrient-poor medium and standard LB yielded similar CFU for *Salmonella* from untreated control mice (3 infections; 1.1 ± 0.1; *P* = 0.19), indicating that slow growth enhanced specifically the survival of enrofloxacin-poisoned *Salmonella* (as in microfluidic devices; Fig. 3g). Further optimization of regrowth conditions might yield even higher CFUs from enrofloxacin-treated mice, but they would still underestimate the number of initially alive *Salmonella* owing to inevitable post-exposure damage by still-bound enrofloxacin (Fig. 3f). Thus, CFU data would remain a confounded readout.

The slow monoexponential SOS kinetics (Fig. 3i) suggested that enrofloxacin damaged *Salmonella* in vivo with low but rather homogenous and constant single-cell probabilities. Thus, the *Salmonella* bulk population, rather than only a minor persister subset, was highly refractory to DNA damage. Against this background of high resilience and massive survival, minor subsets with even superior survival would have limited impact. This was consistent with the only about threefold clearance per day during a 4-day treatment with daily enrofloxacin administration^12^. A *Salmonella* subset with even slower clearance exists in the splenic white pulp. This subset experiences sufficient enrofloxacin exposure, but insufficient local inflammation provides inefficient support for antibiotic clearance. Eventually, inflammation resolves across the entire spleen, resulting in overall eradication failure. By contrast, sustaining inflammation throughout antimicrobial treatment enables eradication from all spleen compartments, confirming the central role of the host immune system and the limited effect of nonreplicating *Salmonella* persisters^12^.

## No effect of auxotrophy and persisters

To explore further the effect of *Salmonella* physiology on antibiotic clearance in vivo, we tested two gene alleles reportedly affecting persister formation. First, we examined a *hisG*^*P69L*^ allele that repairs the histidine auxotrophy of *Salmonella* strain SL1344^38^ used throughout this study. Histidine auxotrophy has been suggested to favour antibiotic tolerance over persistence in vivo, where histidine-limiting conditions are assumed to exist^39^. However, auxotrophic *Salmonella* SL1344 and prototrophic *Salmonella* SL1344 *hisG*^*P69L*^ have undistinguishable replication dynamics and net growth in mice^3^, indicating that host tissues supply sufficient histidine to support the biomass needs of SL1344. Auxotrophic *Salmonella* SL1344 and prototrophic *Salmonella* SL1344 *hisG*^*P69L*^ also exhibited superimposable killing kinetics in enrofloxacin-treated mice (Fig. 1a and Extended Data Fig. 2f; SL1344, survival at 1 h, 5.4 ± 1.8%; 4 h, 2.2 ± 1.2%; SL1344 *hisG*^P69L^: 1 h, 4.3 ± 1.1%; 4 h, 1.5 ± 0.4%;). Thus, the histidine auxotrophy of SL1344 does not affect its antibiotic survival in vivo (Supplementary Note 6).

In addition, we tested a variant of the antitoxin ShpB Q97* (ShpB1) that lacks the last four amino acids, which increases persister frequency in *Salmonella*^40^. *Salmonella hisG*^*P69L*^ *shpB*^Q97*^ exhibited an unaltered growth rate and enrofloxacin MIC in vitro (Extended Data Fig. 1b, MIC = 0.06 mg l^−1^) but survived enrofloxacin exposure in vitro around 30-fold better than wild type (Extended Data Fig. 2g). In mice, *Salmonella hisG*^*P69L*^ *shpB*^Q97*^ showed normal fitness (competitive index versus wild type at day 4 post-infection, 0.79 ± 0.3; *n* = 6; *P* = 0.12, one-sample *t*-test of log-transformed data) and had killing kinetics during enrofloxacin exposure that were undistinguishable from wild type (Extended Data Fig. 2f; Supplementary Note 7). Thus, a mutation increasing the frequency of persisters had no detectable impact on antimicrobial survival in vivo, supporting our model that extensive survival of bulk *Salmonella* minimizes the impact of minor hyper-resilient persister subsets.

In contrast to our findings, it has been reported that infected macrophages trigger *Salmonella* to express toxin–antitoxin modules that arrest growth in a subset of *Salmonella*. These non-replicating persisters maintain an active type 3 secretion system 2 (T3SS-2) which is essential for their survival^41^ and are proposed to cause eradication failures in infected mice receiving daily 300 mg kg^−1^ enrofloxacin (30- to 60-fold more than recommended^42^) for 5 days, starting one day after infection^17^—before disease symptoms appear, which does not reflect typical clinical scenarios (Supplementary Note 8). Some of these data have been difficult to replicate^3,18,43^, the role of non-mutated toxin–antitoxin modules for persister formation is debatable^18,44,45^ (Fig. 1c,d), *Salmonella* does not require T3SS-2 for antibiotic survival in mice^12^, and the evidence for any *Salmonella* surviving such excessive enrofloxacin doses is questionable^3,12,43^ (Supplementary Note 8). Notably, macrophages do not induce a distinct subset of non-replicating *Salmonella* when infected with homogeneously growing *Salmonella* in vivo or in vitro (using *Salmonella* cultured under T3SS-2-inducing conditions to simulate ongoing intracellular replication while preventing rapid macrophage pyroptosis)^3^. A distinct subset of non-replicating intracellular bacteria, largely incapable of resuming growth after being released from host cells, only emerges when macrophages are infected with stationary-phase *Salmonella* cultures^3,17,41^. Stationary cultures are highly heterogeneous^46,47^, and macrophages may solely amplify this pre-existing heterogeneity. In mice, non-replicating *Salmonella* are observed in the first two days following infection with 2 × 10^10^ *Salmonella*^17^, a dose >10^7^ times higher than typically encountered in natural human infections^48^ (Supplementary Note 8). Under more clinically relevant infection and treatment conditions, non-replicating *Salmonella* are rare and have no detectable role in eradication failures^3,12^. Instead, slow bacterial clearance results from slowly replicating *Salmonella*, which respond poorly to antibiotic treatment^3,12,49^.

## Broad post-exposure killing

**Fig. 4:**
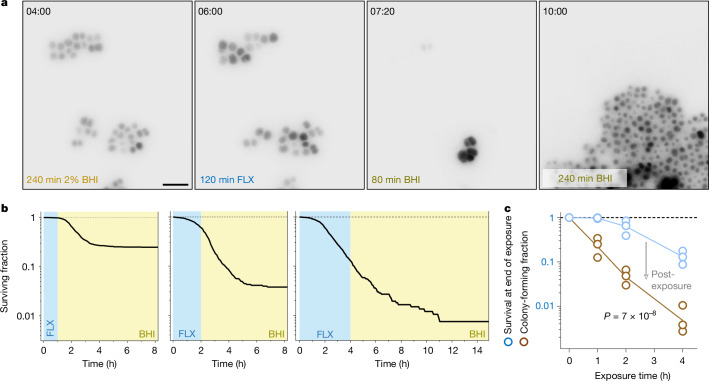
Killing of *S. aureus* by flucloxacillin. **a**, Snapshots of *gfp*-expressing *S. aureus* (inverted fluorescence) before, during and after a 2 h exposure to flucloxacillin (FLX) and switching to antibiotic-free brain–heart infusion (BHI) medium (Supplementary Video 4). Scale bar, 5 μm. **b**, Surviving fractions of *S. aureus* after 1 h (left), 2 h (middle) or 4 h (right) exposure to flucloxacillin followed by switching to BHI medium (937, 745 and 599 cells pooled from three independent experiments). A growing colony originating from a single cell is counted as one survivor. Summary data for individual replicates are shown in **c**. **c**, Surviving fractions of *S. aureus* after 1 h, 2 h or 4 h exposure to flucloxacillin and after switching to antibiotic-free BHI medium. Data from three independent experiments (619, 745 and 917 cells). Lines connect the geometric means. Two-way ANOVA for difference between survival at the end of exposure and colony-forming fractions. The arrow depicts the post-exposure loss of viability. Source data

Our findings with enrofloxacin-treated *Salmonella* raise concerns about interpreting CFU-based killing assays. To test an unrelated pathogen–antibiotic combination, we imaged *gfp*-expressing *Staphylococcus aureus* cells in microfluidic devices during and after exposure to flucloxacillin, a first-line β-lactam antibiotic. *S. aureus* cells started to release their cytosolic contents—indicating death^50^—after approximately 1 h exposure to flucloxacillin (Fig. 4a–c and Supplementary Video 4). After 4 h of exposure, 15% remained alive, but killing continued post-exposure for several hours, consistent with the long-lived transpeptidase inhibition of the covalent penicillin-binding protein–β-lactam complexes^51^. Eventually, only around 0.5% of cells survived and formed microcolonies. The standard CFU plot versus exposure time would suggest monoexponential killing with 30-fold inflated rates compared to actual killing during exposure, which began only after a delay^50^ (Fig. 4c). Thus, CFU again provided a confounded and misleading readout for antibiotic activity.

Previous reports indicate that more than 90% of bacteria that survive initial exposure to fluoroquinolones, β-lactams, aminoglycosides, cidal macrolides, nalidixic acid or trimethoprim may die during regrowth on standard agar plates (Supplementary Note 3), consistent with extensively documented post-antibiotic effects of these and other antibiotics^52^. Specialized regrowth conditions can improve survival by more than 10-fold in most of these cases, but substantial post-exposure death might be unavoidable owing to ongoing damaging effects of remaining target-bound antibiotic (Fig. 3f). As we show here, this post-exposure killing during regrowth can generate artificial biphasic CFU kinetics (Fig. 3d,i), undermining the reliability of the standard persister assay. Additionally, CFU assays may suggest exaggerated killing rates (Figs. 3d,g,i and 4c) and may conflate tolerance (slower killing during exposure) with reduced post-exposure killing (for example, due to slower regrowth; Fig. 3g). Thus, commonly used yet potentially misleading CFU assays should be replaced with real-time, single-cell monitoring of antibiotic action to quantify bacterial killing, tolerance and persistence. For several antibiotic classes, this will require novel reporters for antibiotic-induced damage and viability.

## Discussion

Invasive salmonellosis is a life-threatening disease that requires antimicrobial chemotherapy. The recommended bactericidal antibiotics, fluoroquinolones and cephalosporins, achieve only slow *Salmonella* clearance and are prone to eradication failures, even when the causative *Salmonella* strain tests as susceptible in the laboratory. This poor efficacy is usually attributed to bacterial stress-induced tolerance and persisters. However, our quantitative comparison under physiological conditions suggests that the main reason is nutrient starvation, which restricts *Salmonella* replication in infected tissues^3–5^. *Salmonella* also experiences various stresses in vivo. Although some stresses affect antibiotic survival, their overall effects are limited compared with the dominant role of starvation. Thus, future antibiotic development should mimic nutrient-scarce conditions.

Antimicrobial treatment failures are often attributed to a small subset of non-replicating, hyper-resilient bacteria known as persisters. Persisters are implicated when antibiotic exposure causes an initial rapid decline in CFUs, followed by a slower decrease. *Salmonella* exhibited such a biphasic CFU decline during enrofloxacin treatment in mice. However, we found that standard CFU assays were misleading because enrofloxacin remains bound to its bacterial target gyrase even after washing, continuing to kill the bacteria during regrowth on the plates, which results in low CFU counts even for short treatment intervals. Similar issues may occur with a wide range of other antibiotics. Thus, CFU-based killing assays can provide confounded and misleading results, suggesting non-existing distinct subsets of bacteria with different susceptibilities, underestimating the number of survivors, and misrepresenting their relationship to the bulk population. Using more appropriate single-cell, real-time assays, we found that damage kinetics were rather uniform across the *Salmonella* population and much slower than CFU data suggested. More than 35% of *Salmonella* did not experience any serious damage during 4 h of enrofloxacin exposure, minimizing the influence of rare hyper-resilient persisters. Ceftriaxone also exhibited homogeneously slow killing activity. Thus, the bulk of *Salmonella*, not just a small subset, were difficult to kill. This was consistent with the slow, steady *Salmonella* clearance over several days of treatment with no detectable effect of non-replicating persisters^3,12^.

Our results with two recommended classes of bactericidal antibiotics for treating invasive salmonellosis challenge common assumptions about bacterial resilience and highlight the dominant role of starvation of the entire *Salmonella* population. The limited effects of stresses and persisters observed in our study are consistent with the broad clinical efficacy of short antibiotic treatments for most infections caused by non-resistant bacteria^53^, suggesting limited interference from inflammation-associated stresses and persisters. Longer treatment is required for difficult-to-treat infections such as invasive salmonellosis, tuberculosis, deep-seated *S. aureus* infections and brucellosis. Inefficient drug delivery to infected tissues, biofilms and noncompliant patients may contribute to this problem. These infections also have long incubation periods^54–57^, suggesting slow pathogen growth in human tissues. Quantifying the effects of slow growth, stresses and persisters will require real-time, single-cell assays under physiologically relevant conditions.

## Methods

### Microbiology and molecular biology

*Salmonella* strains used in this study were based on *S. enterica* serovar Typhimurium SL1344 *hisG*^L69P^ *xyl*^38,58^. A histidine-prototrophic derivative SL1344 *hisG*^*P69L*^ and mutants Δ*tisB* and Δ*ecnB* Δ*shpAB* Δ*phD-doc* have been previously described^3^. *Salmonella lexA3* were generated by generalized transduction using P22 phage JS841 Δ*lexA*33::[Cm *lexA3*(Ind−)](sw)^59,60^. *gfp*-expressing strains carried *gfp.mut2* in the chromosomal *sifB* locus that has homogenous high activity in vivo^38,58^. The *recA*^*R29A*^-*mCherry* DNA damage sensor strain carried a pSC101-derivative with the native *P*_recA_ promoter (SL1344 chromosome positions 2,998,612 to 2,998,642) with a modified ribosomal binding site (AGGAA instead of AGGAG) to reduce translation to levels just sufficient for imaging; the entire SL1344 *recA* gene without stop codon and a change of codon 29 from CGT (encoding arginine) to GCG (encoding alanine)^31^; a GGGAGCATC linker encoding Gyl-Ser-Ile^31^; and *mCherry* without the start ATG. The SOS reporter strain carried *mCherry* in the chromosomal *virK* locus^61^ for detecting all bacteria regardless of their SOS response and a pSC101-derivative with a transcriptional fusion of the SOS-inducible *P*_cad_ promoter^36^ of colicin D^62^ to *gfp-ova* coding for a degradable green fluorescent protein variant^61^ (half-life in the range of 30 min). The *P*_cad_ promoter was obtained as synthetic DNA corresponding to bases 6389 to 6675 of pColD-157 (Genbank Y10412.1)^63^ with base 6621 ‘C’ instead of ‘T’ and base 6628 ‘T’ instead of ‘C’^36^ followed by TTAAAAGTCAAAGAGGTGTTTTTGC, containing the ribosomal binding site upstream of *cda* in pColD-CA23^36,62^. *hipA* from *E. coli* BW25113 was mutagenized to obtain *hipA* D88N and expressed in *Salmonella hisG*^P69L^ from the constitutive *P*_ybaJ_ promoter^64^ with a suboptimal ribosomal binding site AAGAG together with *P*_ybaJ_-*timer*^*bac*^ (ref. ^3^) on a pSC101-derivative. *Salmonella hisG*^*P69L*^ *shpB1* was constructed by two consecutive single cross-overs^65^ to change codon 97 from CAA to TAA, resulting in a Q97* mutation, which truncates ShpB by 4 C-terminal amino acids^40^.

*Salmonella* was grown in Lennox LB containing 90 mg l^−1^ streptomycin and 50 mg l^−1^ kanamycin, or in cation-adjusted Mueller–Hinton broth. To mimic in vivo conditions, we cultured *Salmonella* in chemostat medium (100 mM MES, 5 mM KCl, 15 mM NH_4_Cl, 0.5 mM K_2_SO_4_, 1 mM KH_2_PO_4_, 50 μM MgSO_4_, 0.02% casamino acids, 0.02% glycerol, 0.0042% *N*-acetyl-glucosamine, 0.003% glucose, 0.0018% glucosamine, 0.005% histidine, 25 mM NaHCO_3_, pH 5.5; sterile filtered) with a constant stream of 10% O_2_/5 % CO_2_ in mini-chemostats^66^ at various dilution rates (modifications to these conditions are explained in Fig. 2a). Hydrogen peroxide was added 30 min before antibiotic treatment. Growth conditions were maintained for 72 h before addition of antibiotics. For growth in microfluidics devices, we used the same chemostat medium with 1,000-fold decreased levels of all carbon and energy sources.

To determine killing of diluted batch cultures, we diluted overnight cultures 1:1,000 in fresh pre-warmed medium followed by growth for 3 h before antibiotic treatment. For comparing *Salmonella hisG*^*P69L*^ and *Salmonella hisG*^*P69L*^ *shpB*^Q97*^, we grew day cultures for only 1 h before antibiotic treatment as described^40^. To minimize experimental variation in these experiments due to undefined stationary cultures, we standardized the preparation of stationary cultures by inoculating primary overnight cultures from 1-day plate cultures, diluting the cultures after overnight growth 1:1,000 in fresh pre-warmed medium, growing these primary day cultures until exponential phase to an OD_600_ of ~0.2, adjusting them to OD_600_ = 0.1 followed by dilution 1:1,000 in fresh pre-warmed medium, growth for 14 h (secondary defined overnight culture), diluting them again 1:1,000 in fresh pre-warmed medium and treated them with enrofloxacin 1 h later. To remove antibiotics from dilute cultures with minimal loss of *Salmonella* during washing, we mixed the bacterial culture with ~10^9^ CFU diaminopimelic acid-dependent *E. coli* JKe201^65^. These additional bacteria provided sufficient cell mass for a visible and physically stable cell pellet after centrifugation, permitting recovery of >90% of the surviving *Salmonella* after two washing steps to remove the antibiotic. Plating of the resuspended pellets on LB plates without diaminopimelic acid enabled growth of *Salmonella* but not the mixed-in diaminopimelic acid-dependent *E. coli* strain.

We used *S. aureus* strain PROSA28, a strain of clonal complex 45 isolated from a patient with an implant-associated joint infection at University Hospital Basel. We transformed this strain with a pRN11-derivative^67^ carrying a transcriptional fusion of the *P*_pdhABCD_ promoter and *gfp-mut3.1* obtained from plasmid pC183-S3^68^. *S. aureus* was grown in BHI medium containing 10 mg l^−1^ chloramphenicol, or in BHI medium diluted 1:50 with saline (for microfluidics experiments).

### Mouse infections and tissue collection

All animal experiments were approved (license 2239, Kantonales Veterinäramt Basel) and performed according to local guidelines (Tierschutz-Verordnung, Basel) and the Swiss animal protection law (Tierschutz-Gesetz). We estimated sample size by a sequential statistical design. We first infected two to three mice each based on effect sizes and variation observed in our previous studies^5^, and used the results to estimate group sizes for obtaining statistical significance with sufficient power.

Female 10- to 16-week-old BALB/c mice (Charles River Laboratories) were infected by tail-vein injection of ~1,000 CFU *Salmonella* grown to late-log phase in Lennox LB containing 10 mM MgCl_2_ (which increased consistency across experiments). The inoculum size was determined by plating. Intravenously infected mice show similar *Salmonella* growth rates^3^ and *Salmonella* localization in spleen^69^ compared to orally infected mice but exhibit less variation in *Salmonella* tissue loads between individual mice, and thus require fewer experimental animals for detecting differences with the same statistical power. Some mice received at day 5 post-infection an intraperitoneal injection of 5 mg kg^−1^ enrofloxacin or 50 mg kg^−1^ ceftriaxone. For plating, mice were euthanized with carbon dioxide and spleen was homogenized in PBS containing 0.2% Triton X-100. *Salmonella* load was determined by plating. *Salmonella* survival after enrofloxacin administration was determined by comparing flow cytometry counts and corresponding CFU counts of sorted *Salmonella* populations as described^12^. *Salmonella* survival after ceftriaxone administration was determined by plating.

#### Randomization

Control and experimental animals were co-housed. We ensured that litter mate or age-matched and healthy animals with identical sex were used in all experiments. In order to reduce the impact of covariates such as housing and litter size, animals were recruited in a partially randomized manner while taking these factors into account. Control and treated animals were infected with the same inoculum to control for bacterial variation. Data derived from animals were pooled by genotype and/or condition after analyses were completed. Comparisons of isogenic *Salmonella* strains in mice were performed using competitive (mixed) infections to control for variation between experimental animals.

#### Blinding

Data acquisition for drug treatments could not be blinded because the comparisons were made as pre- versus post-drug treatment, the treatment sequence was essential, and often only one drug was applied. For animal welfare reasons, researchers were not blinded to mouse genotype during study and data collection. Specifically, *Slc11a1*^*s*^ mice have to be euthanized at day 4 post-infection to prevent high severity grades, whereas *Slc11a1*^*r*^ mice reach similar bacterial loads only at day 6 post-infection. All other data were collected and analysed without blinding but objectively, using instruments without bias and analysis definitions that were uniformly applied to all data sets.

### Flow cytometry

Spleen was homogenized in ice-cold PBS containing 0.2% Triton X-100. All samples were kept on ice until analysis. Large host cell fragments were removed by centrifugation at 500*g* for 5 min. Relevant spectral parameters were recorded in a FACS Fortessa II operated using BD FACSDIVA V8.0.1 software and equipped with 405 nm, 488 nm and 561 nm lasers (Becton Dickinson), using a threshold on side scatter to exclude electronic noise. We used the following channels: GFP and green TIMER component, excitation 488 nm, emission 502–525 nm; mCherry and orange TIMER component, excitation 561 nm, emission 595–654 nm with red laser (633 nm) switched off; red autofluorescence channel, excitation 488 nm, emission 663–677 nm (the gating strategy for mCherry-*Salmonella* is shown in Extended Data Fig. 2c). *Salmonella* cells were purified from infected spleen homogenates using an Aria IIIu cell sorter (BD Biosciences) using excitation 488 nm and emission channels 499–529 nm (predominantly GFP) and 573–613 (predominantly host autofluorescence) as well as excitation 561 nm and emission 595–617 nm. Defined sample volumes of the sorted *Salmonella* were re-analysed by flow cytometry and plating to determine survival rates^12^. Data were processed with FlowJo 10.6.1 and further analysed with OriginPro 2019 (64-bit) 9.6.0.172.

### Microfluidics experiments

*Salmonella* expressing *recA*^*R29A*^-*mCherry* were grown overnight in chemostat medium, diluted 1:50 in chemostat medium with 100-fold decreased concentrations of carbon and energy sources, grown for 6 h, inoculated in CellASIC ONIX B04A-03 microfluidic plates (Millipore) operated in a CellASIC ONIX Microfluidic System, and grown for 6 h in chemostat medium with 1,000-decreased concentrations of carbon/energy sources (generation time ~3 h), before exposing them to enrofloxacin in the same medium. After various time intervals bacteria were switched back to chemostat medium with 1,000-fold decreased concentrations of carbon/energy sources, and to Lennox lysogeny broth. *gfp*-expressing *S. aureus* were grown overnight in BHI medium diluted 1:50 with saline, diluted 1:50 in fresh medium, grown for 4 h, and inoculated in the microfluidic plates. After growth for 4 h in 1:50 BHI (generation time ~1.5 h), bacteria were exposed to various time intervals to 12 mg l^−1^ flucloxacillin in the same medium, and then switched to normal BHI.

Bacteria were imaged using a Nikon Eclipse Ti2 inverted microscope equipped with Perfect Focus System and Plan Apo 100× Oil Ph3 DM (NA 1.45) objective lens (MRD31905), SPECTRA X light engine (Lumencor), phase contrast (8% light intensity, 200 ms exposure) and a Dualband EGFP/mCherry filter set (Chroma 59022 ET; for *S. aureus*, 470 nm excitation, 4% light intensity, 150 ms exposure; for *Salmonella*, 575 nm excitation, 5% light intensity, 250 ms exposure), a Hamamatsu ORCA-Flash4.0 V3 CMOS camera (C13440-20CU) with pixel size of 65 nm, and NIS-Elements (Nikon). Images were acquired at 1 min (*Salmonella*) or 5 min (*S. aureus*) intervals at 37 °C with 95% humidity controlled by Okolab T-unit (Okolab). Images were analysed with ImageJ 1.53q^70^ using plugins MultiStackReg^71^ and FeatureJ-Laplacian (Smoothing scale 2.0) (developed by E. Meijering).

The decay of undamaged cells in Fig. 3i appeared to occur in three stages based on discontinuities in the first derivative of log-transformed data. We fitted each stage separately with monoexponential decays using GraphPad Prism 9.3.1. Data were further analysed with OriginPro 2019 (64-bit) 9.6.0.172.

### Statistics

Statistical tests were performed with GraphPad Prism 9.3.1 as indicated in the figure legends. We always used two-tailed tests because we were interested to also test for effects that go into the opposite direction to what we might have predicted. CFU counts approximate normal distributions after log transformation^72^, thus permitting parametric test statistics. Multiple linear regression was carried out after log-transforming values for dependent and independent variables (except for pH).

### Reporting summary

Further information on research design is available in the Nature Portfolio Reporting Summary linked to this article.

## Online content

Any methods, additional references, Nature Portfolio reporting summaries, source data, extended data, supplementary information, acknowledgements, peer review information; details of author contributions and competing interests; and statements of data and code availability are available at 10.1038/s41586-024-08506-6.

## Supplementary information

Supplementary Notes Eight supplementary notes that describe previously published data and their contributions to the analyses provided in the main text.

Reporting Summary

Supplementary Video 1 Responses of *Salmonella* to a switch to LB medium. Formation of RecA foci in *Salmonella* growing in nutrient-poor medium that were subsequently shifted to rich lysogeny broth.

Supplementary Video 2 Responses of enrofloxacin-treated *Salmonella* to a switch to LB medium. Formation of RecA foci and induction of SOS responses in *Salmonella* growing in nutrient-poor medium that were exposed to enrofloxacin for 4 h and subsequently shifted to rich lysogeny broth.

Supplementary Video 3 Responses of enrofloxacin-treated *Salmonella* in nutrient-poor medium. Formation of RecA foci and induction of SOS responses in *Salmonella* growing in nutrient-poor medium that were exposed to declining concentrations of enrofloxacin for 7 h and subsequently maintained in nutrient-poor medium.

Supplementary Video 4 Responses of flucloxacillin-treated *S. aureus*. Loss of cytosolic green fluorescent protein in *S. aureus* growing in nutrient-poor medium that were exposed to flucloxacillin and subsequently shifted to nutrient-rich brain–heart infusion.

## Source data

Source Data Fig. 1

Source Data Fig. 2

Source Data Fig. 3

Source Data Fig. 4

Source Data Extended Data Fig. 1

Source Data Extended Data Fig. 2

## Figures and Tables

**Extended Data Table 1:**
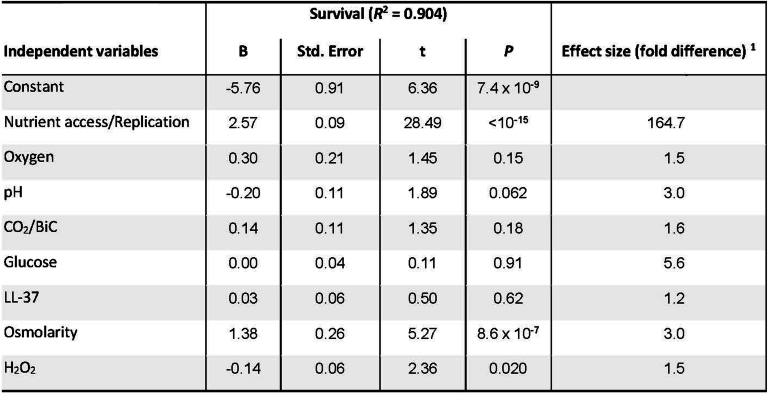
Impact of various stresses on fluoroquinolone activity in vitro

^1^, maximum fold difference in survival within tested range; Std. Error, standard error.

## Data Availability

Data points generated for this study are included in the figures whenever possible. Tabulated data for all figures, videos of the microfluidics experiments and flow cytometry data are available at https://www.ebi.ac.uk/biostudies/studies/S-BSST1727. Source data are provided with this paper.
