# Supplementary material for: Limited impact of Salmonella stress and persisters on antibiotic clearance
Source: Nature. 2025 Feb 5;639(8053):181–9. doi: 10.1038/s41586-024-08506-6 (PMC11882453; doi:10.1038/s41586-024-08506-6)
Supplement: Supplementary file 1 — Eight supplementary notes that describe previously published data and their contributions to the analyses provided in the main text. [file 41586_2024_8506_MOESM1_ESM.pdf]

nature portfolio

https://doi.org/10.1038/s41586-024-08506-6

Supplementary information

Limited impact of *Salmonella* stress and persisters on antibiotic clearance

In the format provided by the authors and unedited

Supplementary Note 1: Impact of various stresses on fluoroquinolone activity *in vitro*

Previous reports have evaluated the impact of environmental conditions on the activity of ciprofloxacin, a fluoroquinolone antibiotic with high similarity to enrofloxacin. The data show that:

- decreasing pH from 7.0 to 5.0 in Mueller-Hinton broth (MHB) increases the minimal inhibitory concentration (MIC) 20-fold for *E. coli* [74];
- increasing osmolarity from ~230 mOsm [75] in lysogeny broth (LB) to ~830 mOsm in LB + 300 mM NaCl increases the MIC for *E. coli* usually 2-fold [76];
- DMEM cell-culture medium with 44 mM $NaHCO_3$ (bicarbonate) under a 5% CO2 atmosphere decreases *Salmonella* MICs 2- to 4-fold (but increases enrofloxacin MICs 2- to 4-fold) compared to Mueller-Hinton broth [77];
- the antimicrobial peptide LL-37 shows weak but significant synergy with ciprofloxacin (fractional inhibitory concentration index 0.5) against *Salmonella* in LB [78];
- the nitric oxide-releasing agent (Z)-1-[N-(2-aminoethyl)-N-(2-ammonioethyl)amino]diazen-1 -ium-1,2-diolate (DETA-NO) does not alter *Salmonella* susceptibility to ciprofloxacin in checkerboard assays [79].

Conditions that are unlikely to occur for *Salmonella* in infected tissues have also been tested:

- Pre-treating *E. coli* in lysogeny broth with 1 mM H_2O_2 before exposure to norfloxacin decreases killing ~10-fold after 1h antibiotic exposure but has no impact after 2h of exposure [80]. This concentration of hydrogen peroxide is several orders of magnitude above the levels that bacteria are likely to experience in natural habitats [81] including inside host phagocytes [69,82,83].
- Restarting growth of stationary-phase *E. coli* in minimal medium by adding 0.4% glucose in presence of 6% oxygen enhances killing by ciprofloxacin ~100-fold [84]. The serum concentration of glucose is ~0.1% and glucose is accessible in only scarce amounts for *Salmonella* in spleen [5].
- Reducing oxygen levels from 6% to undetectable levels (anoxia) during restarting growth of stationary-phase *E. coli* cultures in minimal medium with 0.4% glucose diminishes killing by ciprofloxacin ~100-fold [84]. *Salmonella* are unlikely to experience oxygen levels below 1% in spleen [85] (the focus of this study), because most *Salmonella* reside in an area (the red pulp [12]) with rapid blood circulation and limited local oxygen consumption [86].

Supplementary Note 2: Impact of nitrosative and oxidative stresses *in vivo*

In previous studies, we observed that *Salmonella* subsets in the spleen experience oxidative and nitrosative stresses [69]. Enrofloxacin is ~2-fold less active against *Salmonella* with oxidative stress, and

~1.5-fold more active against *Salmonella* with nitrosative stress compared to unstressed *Salmonella* [12]. *Salmonella* with detoxification defects for reactive nitrogen species experience higher nitrosative stress levels [69], but this has no impact on antibiotic killing [12]. Thus, physiological nitrosative stress may not promote killing itself, but rather represent a marker for microenvironments that enhance enrofloxacin activity. This is consistent with the lack of protection by nitric oxide against fluoroquinolone antibiotics in *Salmonella in vitro* [79] and in cell culture infections [87].

By contrast, recent observations in cell culture suggest that nitrosative stress locks non-replicating *Salmonella* persisters in growth arrest and maintains their resilience against killing by cephalosporins [88]. These results have limited significance for physiological *in vivo* conditions because growth-arrested *Salmonella* are rare *in vivo* [3,12]; most antibiotic survivors originate from slowly replicating instead of growth-arrested *Salmonella* [3,12]; signature proteins for *Salmonella* subsets experiencing nitrosative stress (HmpA, Hcp, YtfE) [69] are not elevated in *Salmonella* cells with sub-average replication rates [3]; and nitric oxide-exposed subsets of *Salmonella* and a detoxification-deficient *Salmonella* mutant experiencing enhanced nitric oxide stress during infection, show no signs of replication slow-down or diminished net growth in mice [69].

Cefotaxime shows stronger anti-*Salmonella* effects in mice lacking iNOS [88]. This has been interpreted as evidence that nitric oxide produced by host iNOS may prevent the awakening of *Salmonella* persisters, allowing the bacteria to survive longer during treatment [88]. However, the interpretation is complicated by two factors: differences in bacterial tissue load before starting cefotaxime treatment and insufficient cefotaxime dosing. In the reported study, iNOS-deficient mice reached ~15 times higher *Salmonella* loads in the spleen two days after oral infection (Fig. S3 in ref. 88). This higher bacterial load in one of the key target organs likely triggered increased body-wide inflammation, resulting in faster bacterial clearance during antimicrobial treatment [12,49]. The cefotaxime dosing was inadequate, as serum drug levels likely stayed above the minimum inhibitory concentration (MIC) only 25% of the time between doses—far below the pharmacological target of 100% for treating serious infections [89]. This calculation is based on the dosing of 150 mg/kg cefotaxime every 12 h [88], an estimated c_{max} in serum of ~75 mg/L [90,91] of which ~50 mg/L would be the active unbound fraction [92], an elimination half-life of ~0.35 h [90,91], and a cefotaxime MIC of 0.15 mg/L for strain *Salmonella* 14028 [93]. Under this suboptimal exposure, drug wash-out kinetics become critical. iNOS-deficient mice may experience slower drug washout due to reduced nitric oxide-induced vasodilation and blood flow [94], potentially leading to higher cefotaxime exposure and enhanced *Salmonella* clearance.

Cefotaxime also shows stronger anti-*Salmonella* effects in mice treated with L-NG-nitroarginine methyl ester (L-NAME), a general inhibitor of nitric oxide synthases. This has been suggested as additional evidence that nitric oxide produced by host iNOS prevents the awakening of *Salmonella* persisters, leading to longer bacterial survival during treatment [88]. However, L-NAME

treatment has pleiotropic effects, making it difficult to attribute its impact on *Salmonella* solely to the inhibition of iNOS. L-NAME also inhibits endothelial nitric oxide synthase, which plays roles in regulating blood pressure, capillary flow, neutrophil infiltration, and reactive oxygen radical production [95]. Studies show that 40 mg/kg L-NAME daily for 4 days (similar to the dosing in [88]) can nearly double mean arterial pressure [96]. Other effects of L-NAME include increased oxidative burst activity in neutrophils [97], elevated lipid peroxidation, reduced plasma glutathione levels, and enhanced platelet activation [98], as well as altered fatty acid metabolism and liver toxicity [99,100]. Because of these pleiotropic effects of L-NAME, its impact on *Salmonella* survival during cefotaxime treatment cannot be traced back to inhibition of iNOS alone.

Supplementary Note 3: Evidence for viability loss of antibiotic-treated bacteria after plating

When bacteria are treated with diverse classes of bactericidal antibiotics and then plated on agar containing rich media, the majority of the initially alive bacteria can lose their viability post-exposure on the plates:

The fluoroquinolones ofloxacin and ciprofloxacin kill non-growing *E. coli* by ~1 log within 5h of exposure based on colony-forming units on LB-agar. However, if treated and washed cells are first incubated for 4h on nutrient-free Gutnick agar without glucose and then transferred to LB-agar, 4- to 8-fold higher colony counts are observed. Thus, 75% to >90% of bacteria that are alive after treatment, lose viability when directly plated on LB-agar [34]. This is consistent with single-cell real-time data suggesting that killing of ciprofloxacin-exposed *E. coli* may occur mostly post-exposure during regrowth on standard media [102]. Likewise, the iron chelator bipyridil when included into LB-agar increases CFU recovery of nalidixic acid-treated *E. coli* ~30-fold, indicating that >95% of initially alive cells lose viability when plated on LB-agar without bipyridil [103].

The killing rate of beta-lactam antibiotics can be overestimated based on CFU data, due to the induction of bacterial filaments and/or spheroplasts [136]. A filament can divide into 20 or more individual cells within 30 to 150 minutes after the antibiotic is removed [137,138]; however, it forms only a single colony on plates, failing to capture the full reproductive potential of filaments. Beta-lactam-induced spheroplasts are initially viable [139] but lyse during regrowth in standard media [140]. Thus, CFU data underestimate the total number of viable cells present at the time of plating, leading to an inflated assessment of the killing rate. This is also supported by the fact that inclusion of bipyridyl and thiourea in LB-agar can increase colony formation of ampicillin-treated *E. coli* 350-fold, indicating that >99% of initially alive cells lose viability on LB-agar without these additions [103].

Macrolides are mostly bacteriostatic (inhibit growth) but not bactericidal (kill bacteria) for *Streptococcus pneumoniae*. However, some macrolides including telithromycin and solithromycin have very low dissociation rates from their target, the ribosome. As a consequence, these drugs stay

bound to the ribosome for up to days after washout, continuously inhibiting translation and ultimately depleting critical proteins to levels insufficient for regrowth, resulting in bacterial death [101]. This bactericidal effect is not occurring during exposure but only much later after washout and plating. Fast-dissociating bacteriostatic macrolides such as erythromycin achieve the same level of translational inhibition during exposure but fail to cause this late impact after washout [101].

Catalase overlay of LB-agar increases colony formation of trimethoprim-treated *E. coli* by 5- to 25-fold compared to LB-agar alone, indicating that up to 96% of initially alive *E. coli* lose viability on LB-agar without catalase [103].

Gentamicin-treated *E. coli* yield 22-fold higher CFU when they are exposed to the uncoupler carbonyl cyanide m-chlorophenylhydrazone (CCCP) prior to plating, indicating that >95% of cells that are alive at the end of exposure lose viability on the plates without CCCP pre-treatment [104]. Importantly, aminoglycosides remain bound to bacteria even after extensive washing, unless the cell envelope is permeabilized by organic solvents (‘irreversible uptake’) [130-132]. Thus, their bactericidal action can continue post-exposure, consistent with their prolonged post-antibiotic effect [132].

Supplementary Note 4: Fundamental differences between cell-culture and mouse infection models

In cell-culture infections, active *Salmonella* cells experience DNA double-strand breaks (DSB) and mount vigorous SOS-responses [105]. This contrasts with the situation in mice, where *Salmonella* SOS-responses are dispensable [60] and remain undetectable unless treated with DNA-damaging antibiotics (Fig. 3h). A requirement for repair of host-induced DSB in mice, particularly in non-growing persisters during antimicrobial treatment, has been suggested [105] based on the attenuation of *Salmonella recA* [60,106]. However, *Salmonella recA* exhibits impaired growth also *in vitro* in lysogeny broth (i.e., without any host-induced damage) [60]. This might be due to the critical roles of RecA-mediated homologous recombination in repairing spontaneously broken DNA replication-forks [107] and spontaneously occurring post-replication gaps [108], in addition to RecA’s involvement in gene regulation [60]. Without further experiments, the *recA* phenotype in mice thus cannot be attributed to host-induced DSBs. It remains also unclear if the ~3-fold diminished survival of *Salmonella recA* after 48h of ampicillin or cefotaxime treatment in mice [105] reflects requirements of non-replicating bacteria. Replicating *Salmonella* are likely to occur under these treatment conditions that include prolonged exposure to sub-MIC drug levels (for cefotaxime. ~75% of the time between doses with exposure levels below MIC, Supplementary Note 2; for ampicillin administered at 150 mg/kg every 12h [105], pharmacokinetics [133] and the MIC of strain 14028 [134] suggest ~77% of the time between doses with exposure levels below MIC). Thus, the ~3-fold lower spleen loads of *Salmonella recA* after antimicrobial treatment for 48h may merely reflect a continuation of the *recA* phenotype of replicating

Salmonella during the preceding acute infection without antimicrobial treatment (~6.5-fold lower loads than *Salmonella* wild-type at 48h post-infection) [105].

The diverging SOS responses observed between cell-culture infections and mice are another example of the many fundamental differences between these experimental models. In mice, most infected host cells harbor only 1 or 2 *Salmonella*, compared to higher intracellular *Salmonella* loads in cell cultures [12,17,109,110]. In mice but not in cell-culture infections, aerobic respiration is essential for *Salmonella* growth [111]. In mice, cardiolipin biosynthesis is dispensable, while it is required in cell-culture infections [112]. Various stress and virulence regulons play different roles in mice and cell-culture infections [113,114] (Supplementary Note 2). As an example, a *Salmonella* mutant with a hypomorphic *rpoS* allele is ~100-fold attenuated in mice but retains wild-type fitness in cell-culture infections [115].

Supplementary Note 5: Calculation of SOS^- and SOS^+ *ex vivo* survivors

At 1h after enrofloxacin administration, 75 ± 7% of *Salmonella* in spleen remained SOS^- (Fig. 3i). Sorting and plating revealed that 6.1 ± 2.0% of this subset gave raise to colonies on LB agar plates (Extended Data Fig. 2e). Multiplying these fractions indicated that 4.6 ± 1.6% of the entire *Salmonella in vivo* population became "SOS^- survivors" (cells with no SOS response at end of exposure that were able to form a colony on LB plates). By contrast, 25 ± 7% of *Salmonella* in spleen were SOS^+ after 1h and sorting/plating revealed that 1.4 ± 0.3% of this subset yielded colonies on LB agar plates. Multiplying the data for SOS^+ indicated that 0.33 ± 0.09% of the entire *Salmonella in vivo* population became "SOS^+ survivors". Comparing 4.6 ± 1.6% SOS^- survivors to 0.33 ± 0.09% SOS^+ survivors revealed that among the survivors, 92 ± 5% originated from SOS^- cells (Fig. 3j; this figure shows means and standard deviations of separate calculations for 6 (1h) and 4 (4h) independently infected mice). Analogous calculations for the 4h treatment revealed that 76 ± 12% of the survivors originated from SOS^- cells (Fig. 3j).

Supplementary Note 6: Influence of histidine auxotrophy on antibiotic survival of *Salmonella*

Michaux et al. [39] suggest that the widely used *Salmonella* strain SL1344 produces misleading results due to its histidine auxotrophy, caused by its dysfunctional *hisG*^{P69} allele. They speculate that in host tissues, limited histidine availability would restrict SL1344's growth, inducing starvation-driven tolerance across the entire bacterial population. This would mask the presence of persister cells. Since histidine auxotrophy is rare among clinical *Salmonella* isolates, SL1344 may be unrepresentative for studying antimicrobial survival.

However, experimental data indicate that SL1344's histidine auxotrophy does not impair its growth *in vivo*. Auxotrophic SL1344 and isogenic prototrophic SL1344 *hisG*^{P69L} (a functional *hisG* allele) show undistinguishable replication rates and net growth in mice, demonstrating that the host provides sufficient histidine to meet the biomass requirements of auxotrophic SL1344 [3], consistent with the broad supply of amino acids available to *Salmonella* in mouse tissues [5]. Facile access to histidine in host tissues is further supported by SL1344's high virulence (i.p. LD_{50} in mice, <20 CFU [38]). Despite citing these studies, Michaux et al. overlook these findings, which contradict their model, and incorrectly cite ref. 38 as evidence for histidine limitation in the host.

Michaux et al. compared SL1344 and SL1344 *hisG*^{P69L} under various *in vitro* conditions, but they only speculate about histidine starvation of SL1344 in mice without providing experimental validation. Instead, Michaux et al. constructed a Δ*hisG* mutant in a different *Salmonella* strain (12023/14028) and observed reduced tissue loads in mice compared to wild-type 12023/14028. They interpret this phenotype as evidence of growth-limiting histidine starvation, neglecting previous studies that demonstrate starvation-independent indirect effects of certain mutations in histidine biosynthesis. Indeed, phenotypes of various histidine auxotrophs in cell-culture infections range from severely attenuated to fully virulent [135]. Michaux et al. incorrectly cite ref. 135 as evidence for histidine limitation in the host, although its discussion section proposes explicitly that intracellular *Salmonella* do not experience a lack of histidine (as demonstrated by normal growth of several histidine auxotrophs) and that the avirulence of a subset of histidine auxotrophs is likely caused by indirect effects [133]. This is consistent with another study showing that 12023/14028 Δ*hisG* (which is similar to Michaux et al.'s mutant) exhibits growth defects in host cells, while other histidine auxotrophs grow normally, confirming sufficient histidine availability [116,117]. The impaired growth of 12023/14028 Δ*hisG* results from overexpression of genes encoding the histidine biosynthesis enzymes HisF and HisH, which can cause a cell division block and filamentation [116,117]. SL1344 displays similar characteristics in cell-culture infections [116] but not in mouse tissues, where <0.1% of SL1344 cells form filaments [12], and restoring functional *hisG* has no significant impact on growth [3].

The regulation of histidine biosynthesis genes is complex [117] and may differ between the newly constructed 12023/14028 Δ*hisG* of Michaux et al. and SL1344, which has been passaged since at least 1980 [38]. Our SL1344 strain's genome differs in seven positions from that of its parental prototrophic strain ST4/74 (all on the chromosome, the three plasmids are unaltered). This includes three synonymous mutations (699,519, C to A, *rlpA*; 1,975,603, G to A, *cheA*; 2,145,516, G to A, *yaaY*), three missense mutations (2,142,870, A to G, *sbcB* V299I; 2,149,950, G to T, *hisC* I106M; 2,147,535, T to C, *hisG* L69P) and a frame-shift (3,708,206, deletion of T; *glpR* at H106). Among these mutations, the missense mutation in *hisC* could potentially modulate *hisG* phenotypes. It does not occur in 14028 (which has an identical *his* operon to that of ST4/74 [58,118,119]). The mutation may impact on the function of the *hisC* gene product histidinol-phosphate, which is involved in histidine

biosynthesis. Moreover, the mutation is located near a regulatory region within *hisC* that is involved in the processing of a stabilized *hisBHAFI* transcript [120]. Besides possible strain differences in *his* regulation, Michaux et al.'s 12023/14028 Δ*hisG* strain may also be affected by unintended secondary mutations, as complementation data are lacking [39]. Regardless of the mechanisms underlying the impaired *in vivo* growth of 12023/14028 Δ*hisG*, this phenotype is irrelevant to SL1344, whose fitness in mice is unaffected by its *hisG*^{P69} allele.

Michaux et al. propose that histidine auxotrophy causes population-wide antimicrobial tolerance in infected tissues, enhancing general *Salmonella* survival and masking the presence of persisters during antimicrobial treatment. However, their own *in vivo* data [39] contradict this hypothesis. During cefotaxime treatment of infected mice, auxotrophic 12023/14028 Δ*hisG* shows no increased tolerance compared to its prototrophic parental strain 12023/14028. Instead, it is effectively eradicated from all analyzed tissues, with even faster clearance rates than 12023/14028 (>8-fold faster in the spleen from day 2 to day 6, >60-fold faster in mesenteric lymph nodes, and >3.5-fold faster in Peyer's patches; Fig. S5 in ref. 39, this effect is invisible in their Fig. 5D and Fig. S4A,B due to the unusual linear scales). These data demonstrate that histidine auxotrophy does not confer antimicrobial tolerance in mice, refuting the model of Michaux et al. Our data show similar clearance of auxotrophic SL1344 and prototrophic SL1344 *hisG*^{P69L} in the spleens of enrofloxacin-treated mice (Fig 1a; Extended Data Fig. 2f). This is consistent with comparable killing of SL1344 by enrofloxacin and ceftriaxone in mice and chemostats with histidine-containing medium (Fig. 1a,b). Thus, SL1344's histidine auxotrophy does not limit its growth in mice and does not enhance antibiotic tolerance or mask persisters *in vivo*.

Supplementary Note 7: Phenotype of *Salmonella shpB1* mutants

Our *Salmonella* SL1344 *hisG*^{P69L} *shpB1* mutant carried a premature STOP codon in *shpB* as in the originally described *shpB1* mutant [40] but no other change. A previously reported *Salmonella* 12023/14028 *shpB1* strain carries the same mutation and an additional scar from lambda red recombineering [39]. This *shpB1* strain exhibits only ~4-fold killing by cefotaxime during 24 h exposure, while the parental wild-type is killed ~100-fold faster. In mice, this *shpB1* mutant grows slower reaching 3-fold lower spleen loads than wild-type at day 2 post-infection. After a six-day treatment with cefotaxime, the total spleen loads decrease by ~10,000 fold. The 12023/14028 *shpB1* strain retains ~4-fold higher loads than its parental wild-type - a small difference compared to its striking *in vitro* phenotype. The increased antibiotic survival in mice may be attributed to persisters [39] or the slower growth of the mutant, and further experiments are necessary to clarify this issue. If persisters do play a (small) role in survival of this anti-toxin mutant *in vivo*, this would not mean that toxins and persisters are critical for survival of wild-type *Salmonella* with non-mutated toxin-antitoxin pairs [45].

Supplementary Note 8: *In vivo* evidence for non-replicating *Salmonella* persisters

It has been suggested that non-replicating persisters dominate among *Salmonella* survivors of antibiotic treatment [121]. To detect non-replicating bacteria, *Salmonella* is induced to express high levels of the green fluorescent protein (GFP) prior to infection. In addition, the bacteria express constitutively mCherry, allowing detection of all *Salmonella*, regardless of GFP levels [109]. As *Salmonella* divides, GFP is diluted by half with each division, diminishing their green fluorescence, whereas non-replicating bacteria retain high GFP and remain brightly fluorescent. Some such GFP-high *Salmonella* are observed in multiple mouse organs during the first two days of infection [7]. After treating the mice for five days with enrofloxacin, ~100 exclusively GFP-high *Salmonella* persist in the mesenteric lymph nodes. This is interpreted as *in vivo* evidence supporting a crucial role for non-replicating persisters in antimicrobial eradication failures. However, methodological caveats, unusual infection and treatment conditions, and an unclear viability read-out limit the physiological relevance of these findings.

The GFP/mCherry method has not yet been assessed for potential fitness costs in *Salmonella* in mice. This would be important because producing high levels of fluorescent proteins impose a metabolic burden on the bacteria, slowing their growth, especially in challenging host environments [122-126]. This raises concerns that the detection method itself might impair *Salmonella* replication in mice.

The inoculum size of 2 x 10^{10} *Salmonella* CFU per mouse used in the persister study [121] is extremely high compared to the doses that humans encounter during natural infections in endemic areas of invasive salmonellosis. Volunteer studies show that a dose of 10^5 *Salmonella* represents most closely natural infections (~50% take, ID_{50}) [48], or only ~1,000 CFU when co-administered sodium bicarbonate neutralizes stomach acid [127] (as in the persister study [121]). The 20,000,000 times higher inoculum used in the persister study likely maximizes the number of disseminating non-replicating persisters. In contrast, mouse infections that mimic human conditions involve doses near the mouse ID_{50} of 10^5 *Salmonella* (without bicarbonate), with only 1-10 bacteria crossing the intestinal barrier and subsequently replicating to high loads in systemic tissues [128]. Under these more physiologically relevant conditions, non-replicating *Salmonella* would remain undetectable in tissues and exert minimal impact on antimicrobial survival.

The potential growth impairment by high GFP/mCherry loads and the high inoculum in the persister study may explain why its findings differ from those using the non-attenuating TIMER^{bac} replication-rate reporter in mice infected with a more moderate *Salmonella* dose (~5 x 10^7 CFU, no bicarbonate; this still elevated dose was used to ensure infection of all mice, limiting the number of required animals). Under these more relevant conditions, non-replicating *Salmonella* are rare and have no detectable impact on antibiotic clearance [3,12].

The persister study used an unusual treatment regime [121] that differed from clinically relevant conditions in both timing and dose. Similar to the extremely high infection dose used, this non-

physiological antimicrobial treatment likely maximizes the number of detectable non-replicating *Salmonella*. Mice were treated with enrofloxacin starting on day 1 post-infection, before disease systems appear. This early treatment ensures a still high proportion of non-replicating *Salmonella* [121] that are exposed to the drug (after high-dose infection). Patients typically receive antibiotic therapy at more advanced stages of infection, after clear disease symptoms have developed. At these later stages, the proportion of non-replicating *Salmonella* becomes small (even after high-dose infection), due to overgrowth by replicating *Salmonella* [121]. Moreover, the increasing inflammation of infected tissues, which plays a significant role in clearing *Salmonella* [12,49], is still weak at early times post-infection [12].

In the persister study, mice were given enrofloxacin at 2g/L in their drinking water [121]. Considering that mice drink ~1.5 mL per day per 10 g of bodyweight [129], this corresponds to a daily dose of ~300 mg/kg. This is 30-60 times higher than the manufacturer's recommended dose for treating infected mice (5 mg/kg once or twice daily; or 10 mg/kg once daily, Bayer Animal Health). Such an excessive dose likely overemphasizes the role of non-replicating bacteria by continuously blocking replication of all *Salmonella* in contrast to more clinically relevant enrofloxacin treatments [3,12].

It remains unclear whether actually any *Salmonella* can survive the excessive enrofloxacin treatment used in the persister study. The viability of non-replicating *Salmonella* in the mesenteric lymph nodes was inferred only indirectly by observing fluorescence changes in bacterial cells after tissue-lysis with 0.3% Triton Tx-100 and 12 h incubation in LB medium [121]. The ~65% drop in GFP fluorescence during this time could reflect GFP dilution due to one to two bacterial divisions, suggesting minimal growth resumption [121]. Alternatively, the decrease in GFP fluorescence could result from cytosolic GFP leaking through a compromised *Salmonella* cell envelope. We detected fluorescent *Salmonella* in the mesenteric lymph nodes under identical treatment conditions, but we were unable to demonstrate any regrowth capability in three independent experiments, even after pre-enrichment in liquid lysogeny broth medium and extended plate incubation [3]. Similarly, another study using the same treatment conditions found no colony-forming *Salmonella* in mesenteric lymph nodes [43], suggesting that the non-replicating *Salmonella* lose viability during the excessive enrofloxacin treatment.

Thus, the only direct experimental evidence supporting a role for non-replicating *Salmonella* persisters in antimicrobial survival within host tissues remains inconclusive. This suggests that non-replicating *Salmonella* persisters may represent a phenomenon relevant primarily in certain cell-culture conditions.

74 Stubbings, W. *et al.* In vitro spectrum of activity of finafloxacin, a novel, pH-activated fluoroquinolone, under standard and acidic conditions. *Antimicrob Agents Chemother* **55**, 4394-4397 (2011).
75 Rothe, M. *et al.* Impact of nutritional factors on the proteome of intestinal *Escherichia coli*: induction of OxyR-dependent proteins AhpF and Dps by a lactose-rich diet. *Appl Environ Microbiol* **78**, 3580-3591 (2012).
76 Everett, M. J., Jin, Y. F., Ricci, V. & Piddock, L. J. Contributions of individual mechanisms to fluoroquinolone resistance in 36 *Escherichia coli* strains isolated from humans and animals. *Antimicrob Agents Chemother* **40**, 2380-2386 (1996).
77 Ersoy, S. C. *et al.* Correcting a fundamental flaw in the paradigm for antimicrobial susceptibility testing. *EBioMedicine* **20**, 173-181 (2017).
78 Sakoulas, G., Kumaraswamy, M., Kousha, A. & Nizet, V. Interaction of Antibiotics with Innate Host Defense Factors against *Salmonella enterica* Serotype Newport. *mSphere* **2**, e00410-00417 (2017).
79 Coban, A. Y. & Durupinar, B. The effect of nitric oxide combined with fluoroquinolones against *Salmonella enterica* serovar Typhimurium in vitro. *Memorias do Instituto Oswaldo Cruz* **98**, 419-423 (2003).
80 Dwyer, D. J. *et al.* Antibiotics induce redox-related physiological alterations as part of their lethality. *Proc Natl Acad Sci U S A* **111**, E2100-2109 (2014).
81 Li, X. & Imlay, J. A. Improved measurements of scant hydrogen peroxide enable experiments that define its threshold of toxicity for *Escherichia coli*. *Free Radic Biol Med* **120**, 217-227 (2018).
82 Winterbourn, C. C., Hampton, M. B., Livesey, J. H. & Kettle, A. J. Modeling the reactions of superoxide and myeloperoxidase in the neutrophil phagosome: implications for microbial killing. *J Biol Chem* **281**, 39860-39869 (2006).
83 Schurmann, N. *et al.* Myeloperoxidase targets oxidative host attacks to *Salmonella* and prevents collateral tissue damage. *Nature microbiology* **2**, 16268 (2017).
84 Gutierrez, A. *et al.* Understanding and Sensitizing Density-Dependent Persistence to Quinolone Antibiotics. *Mol Cell* **68**, 1147-1154.e1143 (2017).
85 Carreau, A., El Hafny-Rahbi, B., Matejuk, A., Grillon, C. & Kieda, C. Why is the partial oxygen pressure of human tissues a crucial parameter? Small molecules and hypoxia. *Journal of cellular and molecular medicine* **15**, 1239-1253 (2011).
86 Vaupel, P., Kallinowski, F. & Okunieff, P. Blood flow, oxygen and nutrient supply, and metabolic microenvironment of human tumors: a review. *Cancer Res* **49**, 6449-6465 (1989).
87 Ronneau, S., Michaux, C., Giorgio, R. T. & Helaine, S. Intoxication of antibiotic persisters by host RNS inactivates their efflux machinery during infection. *PLoS Pathog* **20**, e1012033 (2024).
88 Ronneau, S., Michaux, C. & Helaine, S. Decline in nitrosative stress drives antibiotic persister regrowth during infection. *Cell Host Microbe* **31**, 993-1006.e1006 (2023).
89 Wieringa, A. *et al.* Target attainment and pharmacokinetics of cefotaxime in critically ill patients undergoing continuous kidney replacement therapy. *J Antimicrob Chemother* **77**, 3421-3426 (2022).
90 Sauve, C. *et al.* Efficacies of cefotaxime and ceftriaxone in a mouse model of pneumonia induced by two penicillin- and cephalosporin-resistant strains of *Streptococcus pneumoniae*. *Antimicrob Agents Chemother* **40**, 2829-2834 (1996).
91 Moine, P., Sauve, C., Vallee, E., Bedos, J. P. & Azoulay-Dupuis, E. In vivo efficacy of cefotaxime and amoxicillin against penicillin-susceptible, penicillin-resistant and penicillin---

cephalosporin-resistant strains of *Streptococcus pneumoniae* in a mouse pneumonia model. *Clin Microbiol Infect* **3**, 608-615 (1997).
92 LeFrock, J. L., Prince, R. A. & Leff, R. D. Mechanism of action, antimicrobial activity, pharmacology, adverse effects, and clinical efficacy of cefotaxime. *Pharmacotherapy* **2**, 174-184 (1982).
93 Molina-Quiroz, R. C. *et al.* Exposure to sub-inhibitory concentrations of cefotaxime enhances the systemic colonization of *Salmonella* Typhimurium in BALB/c mice. *Open biology* **5** (2015).
94 Lundberg, J. O. & Weitzberg, E. Nitric oxide signaling in health and disease. *Cell* **185**, 2853-2878 (2022).
95 Wood, K. C. *et al.* Circulating blood endothelial nitric oxide synthase contributes to the regulation of systemic blood pressure and nitrite homeostasis. *Arterioscler Thromb Vasc Biol* **33**, 1861-1871 (2013).
96 Sung, J. H. *et al.* Effect of Lutein on L-NAME-Induced Hypertensive Rats. *Korean J Physiol Pharmacol* **17**, 339-345 (2013).
97 Stehr, S. N. *et al.* N(omega)-nitro-L-arginine methyl ester effects on neutrophil function and bacterial clearance. *Shock* **22**, 180-185 (2004).
98 Freedman, J. E. *et al.* Nitric oxide released from activated platelets inhibits platelet recruitment. *J Clin Invest* **100**, 350-356 (1997).
99 Vos, T. A. *et al.* Differential effects of nitric oxide synthase inhibitors on endotoxin-induced liver damage in rats. *Gastroenterology* **113**, 1323-1333 (1997).
100 Li, B. *et al.* Hypertensive Rats Treated Chronically With N(ω)-Nitro-L-Arginine Methyl Ester (L-NAME) Induced Disorder of Hepatic Fatty Acid Metabolism and Intestinal Pathophysiology. *Front Pharmacol* **10**, 1677 (2019).
101 Svetlov, M. S., Vázquez-Laslop, N. & Mankin, A. S. Kinetics of drug-ribosome interactions defines the cidality of macrolide antibiotics. *Proc Natl Acad Sci U S A* **114**, 13673-13678 (2017).
102 Wong, F. *et al.* Cytoplasmic condensation induced by membrane damage is associated with antibiotic lethality. *Nat Commun* **12**, 2321 (2021).
103 Hong, Y., Zeng, J., Wang, X., Drlica, K. & Zhao, X. Post-stress bacterial cell death mediated by reactive oxygen species. *Proc Natl Acad Sci U S A* **116**, 10064-10071 (2019).
104 Bruni, G. N. & Kralj, J. M. Membrane voltage dysregulation driven by metabolic dysfunction underlies bactericidal activity of aminoglycosides. *Elife* **9**, e58706 (2020).
105 Hill, P. W. S. *et al.* The vulnerable versatility of *Salmonella* antibiotic persisters during infection. *Cell Host Microbe* **29**, 1757-1773.e1710 (2021).
106 Buchmeier, N. A., Lipps, C. J., So, M. Y. & Heffron, F. Recombination-deficient mutants of *Salmonella typhimurium* are avirulent and sensitive to the oxidative burst of macrophages. *Mol.Microbiol.* **7**, 933-936 (1993).
107 Michel, B., Sinha, A. K. & Leach, D. R. F. Replication Fork Breakage and Restart in *Escherichia coli*. *Microbiol Mol Biol Rev* **82** (2018).
108 Cox, M. M. *et al.* Generation and Repair of Postreplication Gaps in *Escherichia coli*. *Microbiol Mol Biol Rev* **87**, e0007822 (2023).
109 Helaine, S. *et al.* Dynamics of intracellular bacterial replication at the single cell level. *Proc Natl Acad Sci U S A* **107**, 3746-3751 (2010).
110 Sheppard, M. *et al.* Dynamics of bacterial growth and distribution within the liver during *Salmonella* infection. *Cell Microbiol.* **5**, 593-600 (2003).
111 Bumann, D. & Schothorst, J. Intracellular *Salmonella* metabolism. *Cell Microbiol* **19** (2017).

112 Cian, M. B. *et al.* Cardiolipin Biosynthesis Genes Are Not Required for *Salmonella enterica* Serovar Typhimurium Pathogenesis in C57BL/6J Mice. *Microbiology spectrum*, e0261721 (2022).

113 Riesenberg-Wilmes, M. R., Bearson, B., Foster, J. W. & Curtis, R., III. Role of the acid tolerance response in virulence of *Salmonella typhimurium*. *Infect.Immun.* **64**, 1085-1092 (1996).

114 Yoon, H., McDermott, J. E., Porwollik, S., McClelland, M. & Heffron, F. Coordinated regulation of virulence during systemic infection of *Salmonella enterica* serovar Typhimurium. *PLoS Pathog* **5**, e1000306 (2009).

115 Wilmes-Riesenberg, M. R., Foster, J. W. & Curtiss, R., III. An altered *rpoS* allele contributes to the avirulence of *Salmonella typhimurium* LT2. *Infect.Immun.* **65**, 203-210 (1997).

116 Henry, T., Garcia-del Portillo, F. & Gorvel, J. P. Identification of *Salmonella* functions critical for bacterial cell division within eukaryotic cells. *Mol.Microbiol.* **56**, 252-267 (2005).

117 Winkler, M. E. & Ramos-Montañez, S. Biosynthesis of histidine. *EcoSal Plus* **3**, 10.1128/ecosalplus. 1123.1126. 1121.1129 (2009).

118 Jarvik, T., Smillie, C., Groisman, E. A. & Ochman, H. Short-term signatures of evolutionary change in the *Salmonella enterica* serovar Typhimurium 14028 genome. *J Bacteriol* **192**, 560-567 (2010).

119 Richardson, E. J. *et al.* Genome sequences of *Salmonella enterica* serovar Typhimurium, Choleraesuis, Dublin, and Gallinarum strains of well- defined virulence in food-producing animals. *J Bacteriol* **193**, 3162-3163 (2011).

120 Alifano, P. *et al.* Processing of a polycistronic mRNA requires a 5' cis element and active translation. *Mol Microbiol* **6**, 787-798 (1992).

121 Helaine, S. & Kugelberg, E. Bacterial persisters: formation, eradication, and experimental systems. *Trends Microbiol* **22**, 417-424 (2014).

122 Wendland, M. & Bumann, D. Optimization of GFP levels for analyzing *Salmonella* gene expression during an infection. *FEBS Lett.* **521**, 105-108 (2002).

123 Rang, C., Galen, J. E., Kaper, J. B. & Chao, L. Fitness cost of the green fluorescent protein in gastrointestinal bacteria. *Can J Microbiol* **49**, 531-537 (2003).

124 Bienick, M. S. *et al.* The interrelationship between promoter strength, gene expression, and growth rate. *PLoS One* **9**, e109105 (2014).

125 Knodler, L. A. *et al.* Cloning vectors and fluorescent proteins can significantly inhibit *Salmonella enterica* virulence in both epithelial cells and macrophages: implications for bacterial pathogenesis studies. *Infect Immun* **73**, 7027-7031 (2005).

126 Bumann, D. *Salmonella* Single-Cell Metabolism and Stress Responses in Complex Host Tissues. *Microbiology spectrum* **7** (2019).

127 Waddington, C. S. *et al.* An outpatient, ambulant-design, controlled human infection model using escalating doses of *Salmonella* Typhi challenge delivered in sodium bicarbonate solution. *Clinical infectious diseases : an official publication of the Infectious Diseases Society of America* **58**, 1230-1240 (2014).

128 Meynell, G. G. The applicability of the hypothesis of independent action to fatal infections in mice given *Salmonella typhimurium* by mouth. *J Gen Microbiol* **16**, 396-404 (1957).

129 Harkness, J. E. & Wagner, J. E. *The biology and medicine of rabbits and rodents 3rd ed.* (Lea & Febiger, 1989).

130 Davis, B. D. Mechanism of bactericidal action of aminoglycosides. *Microbiol Rev* **51**, 341-350 (1987).

131 Wijers, C. D. M. *et al.* Gram-negative bacteria act as a reservoir for aminoglycoside antibiotics that interact with host factors to enhance bacterial killing in a mouse model of pneumonia. *FEMS Microbes* **3**, xtac016 (2022).

132 Lang, M., Carvalho, A., Baharoglu, Z. & Mazel, D. Aminoglycoside uptake, stress, and potentiation in Gram-negative bacteria: new therapies with old molecules. *Microbiol Mol Biol Rev* **87**, e0003622 (2023).

133 van Ogtrop, M. L., Mattie, H., Sekh, B. R., van Strijen, E. & van Furth, R. Comparison of the antibacterial efficacies of ampicillin and ciprofloxacin against experimental infections with *Listeria monocytogenes* in hydrocortisone-treated mice. *Antimicrob Agents Chemother* 36, 2375-2380 (1992).

134 Hossain, M. A. et al. In vitro synergistic potentials of novel antibacterial combination therapies against *Salmonella enterica* serovar Typhimurium. *BMC Microbiol* 20, 118 (2020).

135 Fields, P. I., Swanson, R. V., Haidaris, C. G. & Heffron, F. Mutants of *Salmonella typhimurium* that cannot survive within the macrophage are avirulent. *Proc Natl Acad Sci U S A* **83**, 5189-5193 (1986).

136 Gould, I. M. Pharmacodynamics and the relationship between in vitro and in vivo activity of antimicrobial agents. *J Chemother* 9 Suppl 1, 74-83 (1997).

137 Rolinson, G. N., Macdonald, A. C. & Wilson, D. A. Bactericidal action of beta-lactam antibiotics on *Escherichia coli* with particular reference to ampicillin and amoxycillin. *J Antimicrob Chemother* 3, 541-553 (1977).

138 Libby, J. M. Postantibiotic effect in *Escherichia coli* determined with real-time metabolic monitoring. *Antimicrob Agents Chemother* 42, 78-82 (1998).

139 Lederberg, J. Bacterial Protoplasts Induced By Penicillin. *Proc Natl Acad Sci U S A* 42, 574-577 (1956).

140 Hörnsten, E. G., Nilsson, L. E., Elwing, H. & Lundström, I. Effects of *Escherichia coli* spheroplast formation on assays of H_2 and adenosine triphosphate based ampicillin susceptibility tests. *Diagn Microbiol Infect Dis* 12, 171-175 (1989).
