# Supplementary material for: Limited impact of Salmonella stress and persisters on antibiotic clearance
Source: Nature. 2025 Feb 5;639(8053):181–9. doi: 10.1038/s41586-024-08506-6 (PMC11882453; doi:10.1038/s41586-024-08506-6)
Supplement: Supplementary file 2 — Reporting Summary [file 41586_2024_8506_MOESM2_ESM.pdf]

nature portfolio

Corresponding author(s): Dirk Bumann

Last updated by author(s): Dec 6, 2024

Reporting Summary

Nature Portfolio wishes to improve the reproducibility of the work that we publish. This form provides structure for consistency and transparency in reporting. For further information on Nature Portfolio policies, see our Editorial Policies and the Editorial Policy Checklist.

Statistics

For all statistical analyses, confirm that the following items are present in the figure legend, table legend, main text, or Methods section.

n/a	Confirmed	
☐	☒	The exact sample size (*n*) for each experimental group/condition, given as a discrete number and unit of measurement
☐	☒	A statement on whether measurements were taken from distinct samples or whether the same sample was measured repeatedly
☐	☒	The statistical test(s) used AND whether they are one- or two-sided<br>*Only common tests should be described solely by name; describe more complex techniques in the Methods section.*
☒	☐	A description of all covariates tested
☐	☒	A description of any assumptions or corrections, such as tests of normality and adjustment for multiple comparisons
☐	☒	A full description of the statistical parameters including central tendency (e.g. means) or other basic estimates (e.g. regression coefficient) AND variation (e.g. standard deviation) or associated estimates of uncertainty (e.g. confidence intervals)
☐	☒	For null hypothesis testing, the test statistic (e.g. *F*, *t*, *r*) with confidence intervals, effect sizes, degrees of freedom and *P* value noted<br>*Give P values as exact values whenever suitable.*
☒	☐	For Bayesian analysis, information on the choice of priors and Markov chain Monte Carlo settings
☒	☐	For hierarchical and complex designs, identification of the appropriate level for tests and full reporting of outcomes
☒	☐	Estimates of effect sizes (e.g. Cohen's *d*, Pearson's *r*), indicating how they were calculated

Our web collection on statistics for biologists contains articles on many of the points above.

Software and code

Policy information about availability of computer code

Data collection	Flow cytometry: BD FACSDIVA V8.0.1; Microscopy: VisiView 5.0 software (Visitron Systems)
Data analysis	ImageJ 1.53q 113 using plugins MultiStackReg and FeatureJ-Laplacian; FlowJo 10.6.1; GraphPad Prism 9.3.1; OriginPro 2019 (64-bit) 9.6.0.172

For manuscripts utilizing custom algorithms or software that are central to the research but not yet described in published literature, software must be made available to editors and reviewers. We strongly encourage code deposition in a community repository (e.g. GitHub). See the Nature Portfolio guidelines for submitting code & software for further information.

Data

Policy information about availability of data

All manuscripts must include a data availability statement. This statement should provide the following information, where applicable:

- Accession codes, unique identifiers, or web links for publicly available datasets
- A description of any restrictions on data availability
- For clinical datasets or third party data, please ensure that the statement adheres to our policy

Data points generated for this study are included in the figures whenever possible. Tabulated data for all figures, videos of the microfluidics experiments, and flow cytometry data are available at https://www.ebi.ac.uk/biostudies/studies/S-BSST1727.

Human research participants

Policy information about studies involving human research participants and Sex and Gender in Research.

Reporting on sex and gender	*Use the terms sex (biological attribute) and gender (shaped by social and cultural circumstances) carefully in order to avoid confusing both terms. Indicate if findings apply to only one sex or gender; describe whether sex and gender were considered in study design whether sex and/or gender was determined based on self-reporting or assigned and methods used. Provide in the source data disaggregated sex and gender data where this information has been collected, and consent has been obtained for sharing of individual-level data; provide overall numbers in this Reporting Summary. Please state if this information has not been collected. Report sex- and gender-based analyses where performed, justify reasons for lack of sex- and gender-based analysis.*
Population characteristics	*Describe the covariate-relevant population characteristics of the human research participants (e.g. age, genotypic information, past and current diagnosis and treatment categories). If you filled out the behavioural & social sciences study design questions and have nothing to add here, write "See above."*
Recruitment	*Describe how participants were recruited. Outline any potential self-selection bias or other biases that may be present and how these are likely to impact results.*
Ethics oversight	*Identify the organization(s) that approved the study protocol.*

Note that full information on the approval of the study protocol must also be provided in the manuscript.

Field-specific reporting

Please select the one below that is the best fit for your research. If you are not sure, read the appropriate sections before making your selection.

☒ Life sciences ☐ Behavioural & social sciences ☐ Ecological, evolutionary & environmental sciences

For a reference copy of the document with all sections, see nature.com/documents/nr-reporting-summary-flat.pdf

Life sciences study design

All studies must disclose on these points even when the disclosure is negative.

Sample size	We estimated sample size by a sequential statistical design. We first infected two to three mice each based on effect sizes and variation observed in our previous studies (Steeb et al. 2013) and used the results to estimate group sizes for obtaining statistical significance with alpha < 5% with 90% power.
Data exclusions	No data was excluded.
Replication	We performed all experiments in biological replicates and could observe agreement between the replicates. All experiments were performed at least twice independently and material was collected and processed independently. The exact number of replicates, the exact p-value and the statistical test used to obtain it are indicated in the figures and legends.
Randomization	For in vitro experiments, samples were not randomized but were inoculated from the bacterial culture across all relevant conditions to control for inoculation density and culture properties. Experiments were done in isogenic Salmonella strains.<br>Control and experimental animals were co-housed independent of genotype. We ensured that litter mate or age-matched and healthy animals with identical sex were used in all experiments. In order to reduce the impact of covariates such as housing and litter size, animals were recruited in a partially randomized manner while taking these factors into account. Control and treated animals were infected with the same inocculum to control for bacterial variation. Data derived from animals were pooled by genotype and/or condition after analyses were completed. Comparisons of isogenic Salmonella strains in mice were performed using competitve(mixed) infections to control for variation between experimental animals.
Blinding	Data acquisition for drug treatments could not be blinded because the comparisons were made as pre- vs post-drug treatment, the treatment sequence was essential, and often only one drug was applied. For animal welfare reasons, researchers were not blinded to mouse genotype during study and data collection. Specifically, SLC11A1s mice need to be sacrificed at day 4 post-infection to prevent high severity grades, while SLC11A1r mice reach similar bacterial loads only at day 6 post-infection. Data acquisition and analysis of time-lapse movies could not be blinded because the image sequence clearly revealed the treatment regimen. However rigorous data analysis procedures were implemented to avoid bias. All other data were collected and analyzed without blinding but objectively, using instruments without bias and analysis definitions that were uniformly applied to all data sets.

Reporting for specific materials, systems and methods

We require information from authors about some types of materials, experimental systems and methods used in many studies. Here, indicate whether each material, system or method listed is relevant to your study. If you are not sure if a list item applies to your research, read the appropriate section before selecting a response.

Materials & experimental systems

n/a	Involved in the study
☒	☐ Antibodies
☒	☐ Eukaryotic cell lines
☒	☐ Palaeontology and archaeology
☐	☒ Animals and other organisms
☒	☐ Clinical data
☒	☐ Dual use research of concern

Methods

n/a	Involved in the study
☒	☐ ChIP-seq
☐	☒ Flow cytometry
☒	☐ MRI-based neuroimaging

Animals and other research organisms

Policy information about studies involving animals; ARRIVE guidelines recommended for reporting animal research, and Sex and Gender in Research

Laboratory animals	Mouse, BALB/c, female, age 10-16 weeks, housed at 22°C (-2°C/+3°C), relative humidity of 55 +/- 10%, and a 12h/12h dark/light cycle.
Wild animals	The study did not involve wild animals.
Reporting on sex	Only female mice were used for comparability with previous studies.
Field-collected samples	The study did not involve samples collected from the field.
Ethics oversight	Kantonales Veterinäramt Basel

Note that full information on the approval of the study protocol must also be provided in the manuscript.

Flow Cytometry

Plots

Confirm that:

☒ The axis labels state the marker and fluorochrome used (e.g. CD4-FITC).

☒ The axis scales are clearly visible. Include numbers along axes only for bottom left plot of group (a 'group' is an analysis of identical markers).

☒ All plots are contour plots with outliers or pseudocolor plots.

☒ A numerical value for number of cells or percentage (with statistics) is provided.

Methodology

Sample preparation	Spleen homogenates treated with detergent to liberate intracellular Salmonella or bacterial cultures.
Instrument	BD LSRFortessa, BD FACSAria IIIu
Software	Acquisition: BD FACSDIVA V8.0.1; Analysis: FlowJo 10.6.1;
Cell population abundance	Ex vivo sorted Salmonella samples were 80% pure with host cell fragments as contaminants based on re-analysis with LSR Fortessa. This contamination did not affect downstream analysis of Salmonella.
Gating strategy	Gating strategies were based on fluorescent protein emission and autofluorescence (Extended Data Fig. 4b).

☒ Tick this box to confirm that a figure exemplifying the gating strategy is provided in the Supplementary Information.

March 2021
